# Machine Learning to Predict Quasicrystals from Chemical Compositions

**DOI:** 10.1002/adma.202102507

**Published:** 2021-07-19

**Authors:** Chang Liu, Erina Fujita, Yukari Katsura, Yuki Inada, Asuka Ishikawa, Ryuji Tamura, Kaoru Kimura, Ryo Yoshida

**Affiliations:** ^1^ The Institute of Statistical Mathematics Research Organization of Information and Systems Tachikawa 190‐8562 Japan; ^2^ Department of Advanced Materials Science The University of Tokyo Kashiwa 277‐8561 Japan; ^3^ Department of Materials Science and Technology Tokyo University of Science Tokyo 125–8585 Japan; ^4^ Research and Service Division of Materials Data and Integrated System National Institute for Materials Science Tsukuba 305‐0047 Japan; ^5^ Department of Statistical Science The Graduate University for Advanced Studies Tachikawa 190‐8562 Japan

**Keywords:** approximant crystals, high‐throughput screening, machine learning, materials informatics, quasicrystals

## Abstract

Quasicrystals have emerged as the third class of solid‐state materials, distinguished from periodic crystals and amorphous solids, which have long‐range order without periodicity exhibiting rotational symmetries that are disallowed for periodic crystals in most cases. To date, more than one hundred stable quasicrystals have been reported, leading to the discovery of many new and exciting phenomena. However, the pace of the discovery of new quasicrystals has lowered in recent years, largely owing to the lack of clear guiding principles for the synthesis of new quasicrystals. Here, it is shown that the discovery of new quasicrystals can be accelerated with a simple machine‐learning workflow. With a list of the chemical compositions of known stable quasicrystals, approximant crystals, and ordinary crystals, a prediction model is trained to solve the three‐class classification task and its predictability compared to the observed phase diagrams of ternary aluminum systems is evaluated. The validation experiments strongly support the superior predictive power of machine learning, with the overall prediction accuracy of the phase prediction task reaching ≈0.728. Furthermore, analyzing the input–output relationships black‐boxed into the model, nontrivial empirical equations interpretable by humans that describe conditions necessary for stable quasicrystal formation are identified.

## Graphical abstract

Quasicrystals are long‐range ordered materials that exhibit rotational symmetries disallowed for periodic crystals. Since the first discovery of a quasicrystal in 1984, 100 or so thermodynamically stable quasicrystals have been found, but the pace of the discovery has been slowing down significantly in recent years. Machine‐learning technologies open up the door to the accelerated discovery of new quasicrystals.

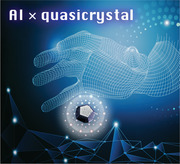

## 1 Introduction

**Figure 1:**
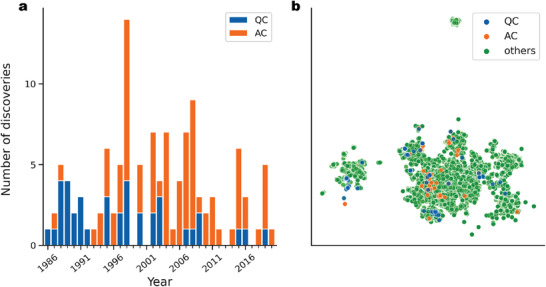
Stable quasicrystals (QC) and approximants (AC) that have been discovered so far. a) Annual trend in the discovery of new thermodynamically stable quasicrystals (blue) and approximant crystals (orange) in aluminum alloys. b) Distribution of the compositional dataset that was visualized onto a 2D space obtained by the UMAP algorithm^[^ ^30^ ^]^ (see the Experimental Section). Stable quasicrystals, approximants, and ordinary crystals are color‐coded by blue, orange, and green, respectively.

This study demonstrates the potential of machine learning to predict stable quasicrystal compositions. Quasicrystals do not have the translational symmetry of ordinary crystals but have a high degree of order in their atomic arrangement. The first quasicrystal was discovered by Shechtman in 1984.^[^ ^1^ ^]^ A few years later, Tsai and his colleagues discovered a series of stable quasicrystals in systems including Al–Cu–Fe, Al–Ni–Co, Al–Pd–Mn, Yb–Cd, and Yb–Cd–Mg.^[^ ^2^, ^3^, ^4^, ^5^, ^6^ ^]^ Since then, 100 or so new stable quasicrystals have been discovered. In the history of quasicrystal research, the discovery of new quasicrystals has unearthed new and interesting phenomena such as anomalous electronic properties,^[^ ^7^, ^8^ ^]^ insulating behaviors,^[^ ^9^ ^]^ valence fluctuation,^[^ ^10^ ^]^ quantum criticality,^[^ ^11^ ^]^ superconductivity,^[^ ^12^ ^]^ and so on. However, the pace of the discovery of new quasicrystals has slowed significantly in recent years. **Figure** **1**a shows the annual trend of new stable quasicrystals found in aluminum alloy systems. From 1986 to 1999, new stable quasicrystals were discovered at a rate of about two per year. On the other hand, in recent years, the frequency of new discoveries has dramatically decreased. This recent trend is mainly due to the fact that no clear guiding principles have been established for the synthesis of new stable quasicrystals. In terms of the stability mechanism of quasicrystals, the Hume‐Rothery rules,^[^ ^13^ ^]^ that is, itinerant valence electron concentration, *e*/*a*, and atomic size factor, have been considered.^[^ ^14^, ^15^ ^]^ However, these are only necessary conditions and are insufficient on their own. Thus, we aimed to accelerate the discovery of new stable quasicrystals by introducing machine learning to the field.

Recently, a wide variety of machine‐learning technologies has been rapidly introduced to materials science. In particular, high‐throughput screening (HTS) across extensive libraries of candidate materials, which typically contain millions or even billions of virtually created candidates, is a promising machine‐learning application. HTS relies on the fast computation of a statistical model that describes physical, chemical, electronic, thermodynamic, and mechanical properties and unobserved structural features as a function of the material. Nowadays, many successful case studies of HTS have been reported. The range of applications is broad, including small organic molecules,^[^ ^16^, ^17^, ^18^ ^]^ polymeric materials,^[^ ^19^ ^]^ inorganic solid‐state materials,^[^ ^20^, ^21^, ^22^, ^23^ ^]^ high‐entropy alloys,^[^ ^24^, ^25^ ^]^ and bulk metallic glasses.^[^ ^26^, ^27^ ^]^ Can HTS based on machine learning also contribute to the discovery of quasicrystals? We seek to answer this question.

The analytical workflow of this study consists of simple supervised learning. The input variable of the model is a chemical composition, which is characterized by a descriptor vector of length 232. As detailed later, the compositional descriptor expresses the content of elements, which is conventionally operated with a predefined set of element features, such as electronegativity and atomic weight.^[^ ^28^, ^29^ ^]^ The output variable is a class label corresponding to one of three structural categories: stable quasicrystal (QC), approximant crystal (AC), and “others,” which includes ordinary periodic crystals. ACs are periodic crystals composed of the similar local structural unit, such as an icosahedral cluster, as the corresponding QCs. Importantly, ACs are formed in nearby compositions of QCs, which means that their stabilization mechanisms are very similar to each other. Hence, for clarification of their common stabilization mechanism, it will be of great advantage to separate ACs from all the other periodic crystals that are termed “others” here. A list of the chemical compositions of known stable quasicrystals, approximants, and ordinary crystals was used as the training data. We systematically evaluated the potential predictability of the proposed machine‐learning model for the three‐class classification problem. Furthermore, virtual screening of all ternary alloy systems containing aluminum and transition elements was conducted for the entire search space. The phase prediction results were compared with 30 experimental phase diagrams extracted from the literature, and the predictability was investigated in detail. The overall accuracy of the phase prediction task reached approximately 0.728. Furthermore, by revealing the input–output landscape inherently encoded in the black‐box model, we identified the law of compositional features relevant to the formation of stable quasicrystalline and approximant crystalline phases. This rule of thumb could be expressed by simple mathematical equations describing a set of compositional features such as the distribution of van der Waals radii of atoms and valence electron concentration. With this study, we take the first step toward enabling the data‐driven discovery of innovative quasicrystals.

## 2 Results

### 2.1 Machine‐Learning Workflow

We used a set of chemical compositions and their class labels for model training. The class labels were QC, AC, and “others” representing types other than the first two. We compiled a list of 80 stable quasicrystals and 78 approximants from the Crystallography of Quasicrystals handbook^[^ ^31^ ^]^ (see Table S1, Supporting Information and Supplementary Data (supporting file 2) for digital data). In addition, the compositions of 10 000 ordinary crystals were randomly extracted from the Materials Project database, which recorded a total of 126 335 crystals.^[^ ^32^ ^]^ We also used 90 crystals from our laboratory data on failed quasicrystal syntheses. These instances form the class “others”. The detailed data preparation procedure is given in the Experimental Section.

**Figure 2:**
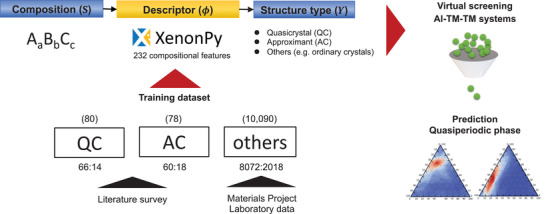
Machine‐learning workflow. The compositional features were encoded into a 232‐dimensional descriptor vector, and a prediction model was created using a random forest classifier. The trained model predicts the class label of a given chemical composition as quasicrystal (QC), approximant (AC), or "others". Model training and testing were performed on the compositional features of 80 known quasicrystals, 78 approximants, and 10 090 ordinary crystals. Finally, we performed HTS across all Al–TM–TM (TM: transition metal) alloys to generate their predicted phase diagrams. The results were compared with experimental phase diagrams obtained from the literature.

The machine‐learning workflow is summarized in **Figure** **2**. The features of a given composition were encoded into a descriptor vector of length 232. The details of the compositional descriptor are described later. The model describes the class label as a function of the descriptor vector of a given composition. We built various models with random forests and neural networks, but since there was no significant difference in prediction performance, this paper presents only the former results. The model training procedure is detailed in the Experimental Section.

For each class, approximately 80% of the total data was randomly selected for training (66, 60, and 8 072 for QC, AC, and “others”, respectively), and the remaining were used as a test set to measure the prediction performance (14, 18, and 2 018 for QC, AC, and “others”, respectively). The configuration of hyperparameters was selected so as to optimize the overall prediction accuracy in the cross‐validation that was looped within the training set (for the list of hyperparameters and their search range, see the Experimental Section). To mitigate the effect of sampling bias on the assessment of predictive performance, we performed 100 random splits of the training and test sets and calculated the mean and variance of the resulting performance metrics.

### 2.2 Representation of Compositional Features

Here, we describe the compositional descriptor. The chemical formula is denoted by $S=S_{c^{1}}^{1}S_{c^{2}}^{2}\cdots S_{c^{K}}^{K}$. Each element of the descriptor vector of length 232 takes the form

(1) $$\begin{array}{c} \phi_{f,\eta }(S)\;\;=\;\;f(c^{1},\ldots ,c^{K},\eta (S^{1}),\ldots ,\eta (S^{K})) \end{array}$$

The notation $\eta \left(S^{k}\right)$ on the right‐hand side denotes a feature quantity of element $S^{k}$, such as the atomic weight, electronegativity, or polarizability. With the function f, the *K* element features $\eta \left(S^{1}\right)$, …, $\eta \left(S^{k}\right)$ with fraction *c* ^1^, …, *c* ^K^ were converted into the compositional feature. For f, we operated with the weighted average, weighted variance, max‐pooling, and min‐pooling as given by

(2) $$\begin{array}{l} \phi_{ave,\eta }(S)\;\;=\;\;\frac{1}{\sum_{k=1}^{K}c^{k}}\sum_{k=1}^{K}c^{k}\eta (S^{k})\\ \phi_{var,\eta }(S)\;\;=\;\;\frac{1}{\sum_{k=1}^{K}c^{k}}\sum_{k=1}^{K}c^{k}{\left(\eta (S^{k})-\phi_{ave,\eta }(S)\right)}^{2}\\ \phi_{\max,\eta }(S)\;\;=\;\;\max\left\{\eta (S^{1}),\ldots ,\eta (S^{K})\right\}\\ \phi_{\min,\eta }(S)\;\;=\;\;\min\left\{\eta (S^{1}),\ldots ,\eta (S^{K})\right\} \end{array}$$

Table S2, Supporting Information provides a list of the 58 element features that were implemented in XenonPy, a Python open‐source platform for materials informatics that we developed.^[^ ^33^ ^]^ The element feature set includes the atomic number, bond radius, van der Waals radius, electronegativity, thermal conductivity, bandgap, polarizability, boiling point, melting point, number of valence electrons in each orbital, and so on.

### 2.3 Generalization Ability of the Model

We predicted the class labels of 2050 test compositions with the 100 trained models. The confusion matrix shown in **Table** **1** and resulting performance metrics suggest that the machine‐learning models were successful in gaining predictive capability. In this analysis, we examined the prediction performance based on three metrics: recall, precision, and F‐value. These metrics quantified the predictive performance for each class c of QC, AC, and “others” according to

(3) $$\begin{array}{l} \text{Recall}(c)\;\;=\;\;\frac{\text{TP}(c)}{\text{TP}(c)+\text{FN}(c)}\\ \text{Precision}(c)\;\;=\;\;\frac{\text{TP}(c)}{\text{TP}(c)+\text{FP}(c)}\\ F_{1}(c)\;\;=\;\;2\;\cdot \;\frac{\text{Recall}(c)\cdot \text{Precision}(c)}{\text{Recall}(c)+\text{Precision}(c)} \end{array}$$

TP(c) denotes the number of true positives when label c is treated as positive and the other two classes as negative, and FN(c) and FP(c) represent a false negative and false positive, respectively. Thus, the recall rate represents the fraction of compositions with true class label c that could be predicted as c, whereas the precision represents the fraction of compositions predicted as label c that were actually label c. There is a tradeoff between the recall and precision rates. F_1_(c) is the harmonic mean of the recall and precision.

**Table 1:** Prediction performance for the three‐class classification problem of stable quasicrystals (QC), approximants (AC), and “others”. The left table is the confusion matrix, and the right table reports the per‐class recall, precision, and F_1_ metrics. The performance metrics were averaged over 100 different bootstrap sets, and the numbers in parentheses represent the standard deviations. In Table S3, Supporting Information, we also show the performance evaluation results after eliminating the aluminum‐containing compositions from the test instances

		**Predicted class**							
		**QC**	**AC**	**Others**			**Recall**	**Precision**	**F**_1_
**True class**	**QC**	9.63 (1.641)	3.24 (1.342)	3.13 (1.189)		**QC**	0.602 (0.103)	0.722 (0.090)	0.650 (0.076)
	**AC**	3.11 (1.555)	9.73 (1.805)	3.16 (1.573)		**AC**	0.608 (0.113)	0.731 (0.089)	0.658 (0.088)
	**Others**	0.76 (0.896)	0.42 (0.619)	2016.82 (1.024)		**Others**	0.999 (0.001)	0.997 (0.001)	0.998 (0.001)

The precision and recall for the prediction of the class “others” reached 0.997 and 0.999, respectively. This means that almost perfect predictions were achieved for the binary classification of QC/AC as a merged class versus “others”. On the other hand, the precision and recall were 0.722 and 0.602 for QC and 0.731 and 0.608 for AC, respectively. Although the classification performance was slightly lower than that in the prediction of the class “others”, the trained models exhibit the generalized ability to identify chemical compositions that could potentially generate stable quasicrystals and approximant crystals.

### 2.4 Phase Prediction of Ternary Alloy Systems

Of the 100 models shown above, the model that achieved the highest prediction accuracy was selected, and high‐throughput virtual screening of all composition spaces was performed on a total of 1 080 systems of Al–TM[4,5]–TM[4,5] (TM: transition metal) and Al–TM[4,5]–TM[6], where the numbers in square brackets denote the periods of the transition elements. In addition, we added a set of non‐transition‐metal elements {Mg, Si, Ga, Ge, In, Sn, Sb} in place of TM[4,5] and {Tl, Pb, Bi} in place of TM[6]. With a given model, the class probability of QC, AC, or “others” was calculated for a given chemical composition. For each composition, we standardized its fractions into relative proportions. A ternary phase diagram was gridded with 20 301 points by dividing the interval of the composition ratio from 0 to 1 by 200 equally spaced grid points. A label exhibiting the maximum probability was assigned to each grid point in the diagram. In this way, stable quasicrystalline and approximant phases were predicted. Using this screening process, quasicrystalline phases were predicted to exist in 185 systems, which would be an overestimate. Notably, in 136 of the 185 systems, the predicted quasicrystalline and approximant phases coexisted in neighboring regions of the same diagram. This result is highly consistent with experimental observations, which we give examples of later.

**Figure 3:**
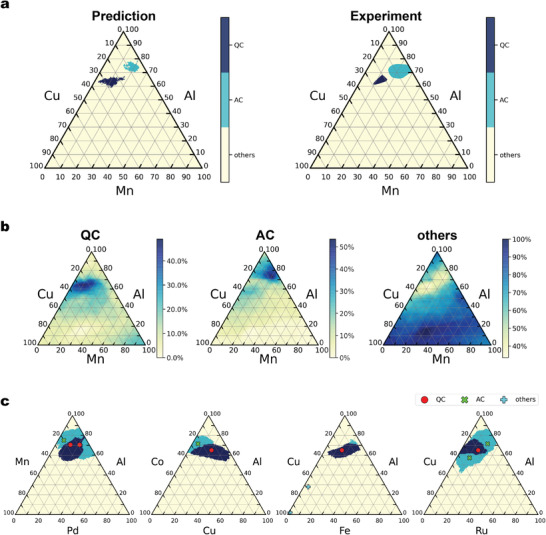
Phase prediction of the Al–Mn–Cu system. a) Predicted phase diagram (left) and experimental phase diagram (right) of the Al–Mn–Cu system. The three colors denote the stable quasicrystalline phase (QC), approximant phase (AC), and “others”. Despite the lack of training instances for Al–Mn–Cu, the model successfully predicts the unseen stable quasicrystalline and approximant crystalline phases. b) Heatmap display of the predicted class probability of QC, AC, and “others” for the Al–Mn–Cu system. c) In order to observe the training instances relevant to the model decision making, we examined the distribution of training instances in the four ternary systems closest to Al–Mn–Cu.

We verified the validity of the predicted phase diagrams based on the experimental stable quasicrystal and approximant phase regions of the 30 systems that were extracted from the literature.^[^ ^34^, ^35^, ^36^, ^37^, ^38^, ^39^, ^40^, ^41^, ^42^, ^43^, ^44^, ^45^, ^46^, ^47^, ^48^, ^49^, ^50^, ^51^, ^52^, ^53^, ^54^, ^55^, ^56^, ^57^, ^58^ ^]^ We found 198 papers published by Prof. Grushko's group, which include ternary phase diagrams of Al–transition elements encompassing 64 unique alloy systems. Excluding the systems containing the 80 stable quasicrystal and 78 approximant compositions used for training, the remaining 30 systems were used for performance evaluation. Figure S2, Supporting Information displays all the predicted and experimental phase diagrams, and **Figure** **3** shows an example. With a given classifier, the class probability of forming quasicrystals, approximants, or others was drawn on the phase diagram of Al–Cu–Mn.^[^ ^52^ ^]^ To evaluate the prediction performance, the agreement between the three class probabilities and the experimental quasicrystalline and approximant phase regions was investigated. For each ternary system, $G_{c_{\exp}}$ denotes the set of all grid points in experimental phase $c_{\exp}\in \{\text{QC},\text{AC},\text{others}\}$ in a diagram. Using the trained model, we calculated the mean probability $p(Y=c|G_{c_{\exp}})$ for each $c_{\exp}$ and c∈{QC,AC,others} by

(4)

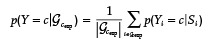


**Figure 4:**
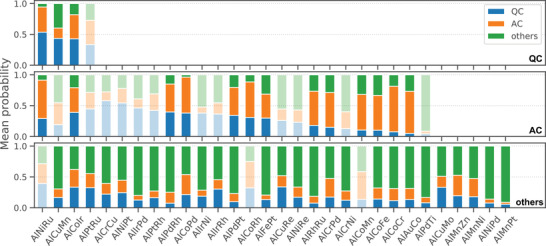
Prediction performance for the 30 different Al–TM–TM systems. The mean class probability was calculated in each of the experimental phase regions (top: QC, middle: AC, and bottom: “others”) using our trained random forest classifier. The bar plots shown in a transparent color represent phases where class label prediction based on maximum probability failed.

$p\left(Y_{i}=c{|S}_{i}\right)$ denotes the predicted probability that class label $Y_{i}$ of composition $S_{i}$ with $i\in G_{c_{\exp}}$ is equal to c. The probability values were averaged over a given phase with grid points $i\in G_{c_{\text{expt}}}$. If $p(Y=c{|G}_{c_{\text{expt}}})$ reaches a maximum at $c=c_{\exp}$, the prediction is correct. The prediction performance across the 30 alloy systems is summarized in **Table** **2**. In addition, the mean probability of each class with respect to the three different phases in the 30 systems is displayed in **Figure** **4**.

**Table 2:** Phase prediction performance for the 30 Al–TM–TM (TM: transition metal) alloy systems

		**Predicted class**							
		**QC**	**AC**	**Others**			**Recall**	**Precision**	**F**_1_
**True class**	**QC**	3	1	0		**QC**	0.750	0.333	0.462
	**AC**	5	13	7		**AC**	0.520	0.813	0.634
	**Others**	1	2	27		**Others**	0.900	0.794	0.844

The results showed a similar trend to the performance metrics from the previously discussed composition‐level evaluation (Table 1). The precision and recall were 0.333 and 0.750 for quasicrystals, 0.813 and 0.520 for approximants, and 0.794 and 0.900 for others, respectively. The overall prediction accuracy reached 0.728. As shown in Figure 4, the number of cases in which a quasicrystalline phase region was misclassified as an approximant was 1/4, and the number of cases in which an approximant phase was misclassified as a quasicrystal was 5/25. On the other hand, the other regions, including the ordinary crystalline phases, were almost completely predictable. Although the misclassification rate for quasicrystalline and approximant phases increased slightly, the trained model was found to have sufficient predictive power to be useful.

Although the misclassification rate between quasicrystalline and approximant phases was slightly high, we concluded that the model is more or less capable of identifying compositional regions of quasicrystals and approximant crystals. As illustrated in Figure 3 showing the Al–Mn–Cu phase diagram and its prediction results, in many cases, the model adequately captured not only the positional features of the quasicrystalline and approximant phases but also their contour shapes (see also Figure S1, Supporting Information for all results). Interestingly, despite the lack of any training instances from the Al–Mn–Cu system, the model successfully predicted the two true phase regions. In order to identify the instances in the dataset on which the model relied in the training process, four other systems with the closest compositional patterns to Al–Mn–Cu were selected, and the distribution of the training data was examined (Figure 3c). The compositional closeness was evaluated based on the Euclidean distance of the normalized 232‐dimensional compositional descriptor. Simple pattern matching based on the similarity of the input and output to the training data never predicted the positional and geometric features of the quasicrystalline and approximant phases in the Al–Mn–Cu phase diagram. Thus, the model involves a higher‐order recognition mechanism than simple nearest‐neighbor matching.

### 2.5 Hume‐Rothery's Law Autonomously Learned by Machine Learning

Notably, it was found that the trained models learned Hume‐Rothery's electron concentration law,^[^ ^13^ ^]^ which is one of the most widely applied empirical rules regarding the formation of stable quasicrystalline alloys. In 1990, Tsai et al. discovered a series of thermally stable quasicrystals in the Al–Cu–TM and Al–Pd–TM systems.^[^ ^2^, ^3^, ^4^ ^]^ In a subsequent study, the discovered stable quasicrystals were found to obey Hume‐Rothery's electron concentration law on the average itinerant valence electron number *e*/*a*.^[^ ^14^ ^]^

**Figure 5:**
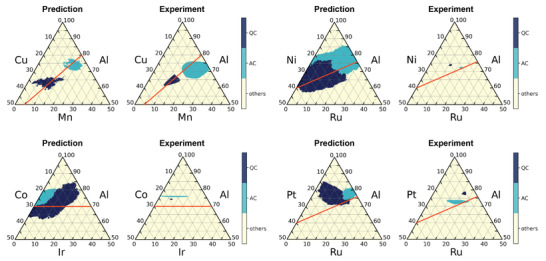
Predicted and experimental phase diagrams of four ternary alloy systems. The orange lines indicate the Hume‐Rothery rule of valence electron concentration with *e*/*a* = 1.8.

**Figure** **5** shows the predicted and experimental phase diagrams for four of the 30 evaluated alloy systems as discussed above. In each diagram, the line where the average itinerant valence electron number follows *e*/*a* = 1.8 is overlaid (see ref. [59] for details on the calculation of *e*/*a*). Surprisingly, in all the systems, the straight lines overlap with the predicted and true regions of quasicrystals and approximant crystals. In the 30 ternary alloy systems discussed above, the straight line completely overlapped with the predicted regions in 26 systems (Figure S1, Supporting Information). Note that our compositional descriptors do not include *e*/*a* values; this widely known empirical rule occurred via the nonlinear mapping of our descriptors. If we can comprehensively extract such implicit rules inherent to the trained machine‐learning model, we could obtain hypothetical insights on the formation rules or mechanisms of quasicrystalline phases.

### 2.6 Why Can the Model Predict Quasicrystals?

To determine on what basis the model determines structural classes, we analyzed the predicted class labels $\left\{Y_{i}|i=1,\ldots N\right\}$ in relation to the 21 925 080 hypothetical compositions $\left\{S_{i}|i=1,\ldots ,N\right\}$ (*N* = 21 925 080) that were used in the HTS of the entire composition space of the 30 aluminum alloys. The model determined mathematical map $Y=f\left(S\right)$ between predicted label Y and descriptor vector $\phi (S)=(\phi_{1}(S),\ldots ,\phi_{232}(S))\in {\mathbb{R}}^{232}$. First, we examined the degree of relevance of each descriptor element $\phi_{h}\left(S\right)$ (h=1,...,232) with respect to predicted Y. As a quantitative measure of relevance, we applied the maximal information coefficient (MIC), a widely used measure of statistical independence (linear and nonlinear correlation) between two variables.^[^ ^60^ ^]^ Using the dataset $\left\{(Y_{i},\phi_{h}(S_{i}))|i=1,\ldots ,N\right\}$, which was produced from the black‐box machine‐learning model, we estimated the joint distribution $P\left(Y,\phi_{h}\left(S\right)\right)$ and marginal distribution $Q\left(Y,\phi_{h}\left(S\right)\right)=P\left(Y\right)P\left(\phi_{h}\left(S\right)\right)$, where the latter assumes independence between Y and $\phi_{h}\left(S\right)$. The MIC evaluates the statistical independence of the *h*th descriptor $\phi_{h}\left(S\right)$ and output Y by measuring the discrepancy between $P\left(Y,\phi_{h}\left(S\right)\right)$ and $Q\left(Y,\phi_{h}\left(S\right)\right)$. The Kullback–Leibler divergence, which is equivalent to the mutual information between $\phi_{h}\left(S\right)$ and Y, was employed for the MIC evaluation, and an adaptive binning algorithm was applied to approximate the two probability distributions by generating histograms.

**Table** **3** shows the top 20 most relevant descriptors as examples, which suggest that the weighted averages of the van der Waals radius, electronegativity, and first ionization energy are highly relevant to the basis of the model decision making process. The most relevant descriptor, that is, the weighted average of the van der Waals radius, is consistent with the Hume‐Rothery rules, where the atomic size factor is considered to contribute to the stability mechanism of icosahedral quasicrystals. In addition, Table 3 shows the within‐class mean and within‐class variance of the subset of $\left\{\phi_{h}(S_{i})|i=1,\ldots ,N\right\}$ belonging to each {QC,  AC,  others}. Descriptors with larger discrepancies in the within‐class means and smaller within‐class variances are interpreted as having a high degree of separation between classes and thus a high degree of relevance to the output class label. Most of the listed relevant descriptors exhibited significantly large between‐class separations in terms of QC/AC versus “others” or QC versus AC.

**Table 3:** The 20 most relevant descriptors in the classification task for the 30 Al–TM–TM alloy systems. The first column shows the descriptor ID (upper) and name (lower) in XenonPy. The prefixes “ave” and “var” in the descriptor ID represent weighted average and weighted variance types, respectively. The last four columns show the within‐class means of the QC, AC, “others”, and QC/AC‐merged groups. The within‐class variances (converted to standard deviations) are reported in parentheses

**Descriptor information**	**MIC**	**QC**	**AC**	**Others**	**QC/AC**
**ave:vdw_radius_uff**					
Van der Waals radius from the UFF [pm]	0.43	409.05 (3.37)	406.49 (6.81)	382.30 (40.66)	406.59 (6.73)
**ave:en_ghosh**					
Ghosh's scale of electronegativity	0.42	0.15 (0.00)	0.15 (0.01)	0.16 (0.02)	0.15 (0.01)
**ave:first_ion_en**					
First ionization energy [eV]	0.41	6.49 (0.09)	6.53 (0.17)	6.84 (0.58)	6.53 (0.17)
**ave:mendeleev_number**					
Mendeleev's number	0.41	75.94 (0.36)	75.86 (1.47)	73.32 (4.33)	75.87 (1.45)
**ave:specific_heat**					
Specific heat at 20 °C [J g^−1^ mol^−1^]	0.40	0.74 (0.02)	0.73 (0.04)	0.66 (0.15)	0.73 (0.04)
**ave:num_p_valence**					
Number of filled p valence orbitals	0.40	0.71 (0.06)	0.73 (0.05)	0.57 (0.25)	0.73 (0.05)
**ave:num_p_unfilled**					
Number of unfilled p valence orbitals	0.40	3.53 (0.30)	3.63 (0.24)	2.85 (1.24)	3.63 (0.24)
**ave:heat_capacity_mass**					
Specific heat capacity at STP [J K^−1^ mol^−1^]	0.40	0.74 (0.02)	0.73 (0.04)	0.66 (0.15)	0.73 (0.04)
**ave:covalent_radius_cordero**					
Covalent radius by Cerdero et al. [pm]	0.39	126.06 (1.19)	126.38 (2.13)	129.51 (6.31)	126.37 (2.10)
**ave:vdw_radius**					
Van der Waals radius [pm]	0.37	189.51 (0.55)	190.67 (1.81)	193.80 (6.33)	190.63 (1.79)
**ave:gs_energy**					
Ground state energy at *T* = 0 K [eV atom^−1^]	0.37	−4.57 (0.18)	−4.69 (0.28)	−5.19 (1.12)	−4.68 (0.28)
**ave:thermal_conductivity**					
Thermal conductivity at 25 °C [W m^−1^ K^−1^]	0.36	221.35 (21.74)	201.23 (13.96)	170.72 (60.68)	201.99 (14.83)
**ave:covalent_radius_slater**					
Covalent radius by Slater [pm]	0.35	127.92 (1.19)	128.08 (0.93)	130.40 (3.38)	128.08 (0.94)
**ave:period**					
Period in periodic table	0.35	3.40 (0.06)	3.52 (0.19)	3.73 (0.55)	3.52 (0.19)
**var:num_p_valence**					
Number of filled p valence orbitals [pm]	0.34	0.20 (0.02)	0.20 (0.02)	0.18 (0.07)	0.20 (0.02)
**ave:num_d_valence**					
Number of filled d valence orbitals [pm]	0.34	2.30 (0.60)	2.08 (0.52)	3.15 (1.90)	2.09 (0.52)
**ave:heat_capacity_molar**					
Molar heat capacity at STP [J K^−1^ mol^−1^]	0.34	24.44 (0.10)	24.47 (0.14)	24.81 (0.58)	24.47 (0.14)
**ave:density**					
Density at 295 K [g cm^−3^]	0.34	4.94 (0.32)	5.55 (1.24)	6.70 (3.35)	5.53 (1.23)
**var:num_p_unfilled**					
Number of unfilled p valence orbitals	0.34	5.09 (0.60)	4.91 (0.48)	4.60 (1.77)	4.91 (0.49)
**ave:hhi_p**					
Herfindahl–Hirschman index (HHI) production values	0.33	1810.99 (242.60)	2106.51 (274.67)	2196.88 (706.35)	2095.30 (279.22)

**Figure 6:**
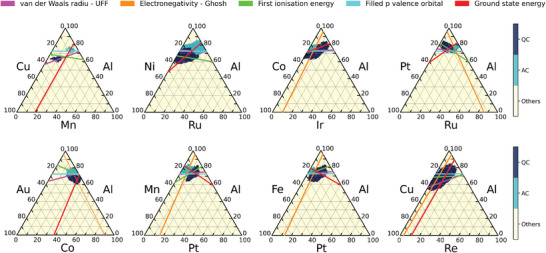
Five rules for the formation of QC/AC phases proposed by machine learning. The rules are represented by straight lines on the predicted phase diagrams of the eight systems. Each line represents a condition set $C_{h}$ describing the weighted average of the van der Waals radius, electronegativity, first ionization energy, number of filled *p* valence orbitals, or energy per atom in the *T* = 0K ground state that is imposed on the compositional formula.

Only listing highly relevant descriptors is not enough to clarify the basis of the model decision making process. Instead, we want to derive an explicit empirical equation, such as the rule of *e*/*a* = 1.8 for itinerant valence electron concentration. In this study, we focused on the binary classification task of discriminating between merged QC/AC and “others”. We calculated the within‐class mean $m_{h}$ for the QC/AC group from the observed $\left\{\phi_{h}(S_{i})|i=1,\ldots ,N\right\}$ with their predicted Y=QC or AC. It is expected that the model places a high classification probability *p* (Y∈{QC,  AC}|S) on any composition ratio S that satisfies exactly or approximately $\phi_{h}\left(S\right)=m_{h}$. For example, in the case where S is a ternary system $S_{{\hat{c}}^{1}}^{1}S_{{\hat{c}}^{2}}^{2}S_{{\hat{c}}^{3}}^{3}$ and the descriptor $\phi_{h}\left(S\right)$ is of the weighted average type, we could identify the composition ratio $\left({\hat{c}}^{1},{\hat{c}}^{2},{\hat{c}}^{3}\right)$ that approximately satisfies the following condition:

(5) $$\begin{array}{c} C_{h}=\left\{({\hat{c}}^{1},{\hat{c}}^{2},{\hat{c}}^{3})|\sum_{i\;=\;1}^{3}{\hat{c}}^{i}\eta (S^{i})=m_{h},\sum_{i\;=\;1}^{3}{\hat{c}}^{i}=1,{\hat{c}}^{i}\geq 0\;(\forall i)\right\} \end{array}$$

where ${\hat{c}}^{i}$ denotes the normalized fraction and $\eta \left(S^{i}\right)$ is the feature value of element $S^{i}$. Without any loss of generality, $C_{h}$ can be defined for any system or other descriptor type such as the weighted variance. Here, we focused on the weighted average descriptors of the van der Waals radius (“ave:dw_radius_uff”), Ghosh's scale of electronegativity (“ave:en_ghosh”), first ionization energy (“ave:first_ion_en”), number of filled *p* valence orbitals (“ave:num_p_valence”), and energy per atom in the *T* = 0K ground state calculated by density functional theory (“ave:gs_energy”) among the highly relevant descriptors listed in Table 3. Then, we overwrote each $C_{h}$ on the predicted phase diagrams for the 30 alloy systems. **Figure** **6** illustrates eight selected phase diagrams (see also Figure S2, Supporting Information for the results of all 30 systems). In almost all systems, the straight lines $C_{h}$ conditioned by the five relevant descriptors passed through the predicted QC and AC phase regions. Note that each $C_{h}$ is one of the necessary conditions for the formation of QC and AC phases. The intersection of these conditions defines a set of empirical equations for determining the compositional ratio that forms a quasicrystal or approximant.

In this way, the implicit rules extracted by the machine‐learning algorithm can be encoded in a simple mathematical form interpretable by humans. By accumulating such empirical rules, performing verifications, and pursuing theoretical explanations, we can gain new scientific knowledge. It is important to note that the empirical equations described here are subject to various restrictions in terms of their applicable domains. Specifically, they may be local rules obtained from the input–output of the trained model for ternary alloys of Al–TM–TM and thus would not be generally applicable to other systems. There must be many other implicit rules to discover from the trained model, and thus it is important to exhaustively extract these implicitly encoded rules and clarify their range of application at the same time.

## 3 Discussion

This study demonstrated the predictive power of machine learning for the identification of candidate compositions to form quasicrystalline and approximant alloys. The problem was formulated rather simply as a supervised learning task of classifying any given composition into one of three kinds of material structures: quasicrystals, approximants, and others representing types other than the first two. Although supervised learning was conducted with a conventional random forest classifier, the model trained only on a list of known compositions reached a high prediction accuracy. In a binary classification task of predicting a combined quasicrystal/approximant class versus others, the precision and recall reached 0.997 and 0.999, respectively. In addition, it was confirmed that the model can discriminate between quasicrystals and approximants, although the accuracy is slightly lower. If this approach can be used to narrow candidate compositions for forming quasicrystals and approximants, the efficiency of related materials searches would be greatly improved.

The predictability of machine learning for this task has been proven. However, before putting the approach into practice, some remaining questions need to be answered. The first is why the machine‐learning models can predict the compositions of quasicrystals. In the present study, we evaluated the relevance of the descriptors based on the MIC metric and narrowed the total to five descriptors that are strongly involved in the model decision making process. According to the identified descriptors, we derived five empirical equations with high interpretability that are presumed to be necessary conditions for the formation and hence the stability of quasicrystals and approximants. Importantly, these newly identified conditions will lead to the long‐sought and heretofore unclear guiding principles for the synthesis of new quasicrystals, thereby opening the door to a deeper understanding of quasicrystal stability as a central issue in condensed matter physics. Many other implicit rules are still embedded in the learned model. By identifying the comprehensive set of rules encoded in the black‐box machine‐learning model, we will piece together the puzzle and record statements as rules of thumb for materials science.

The other remaining question concerns the applicable domains of these machine‐learning models. Most of the quasicrystals found thus far are binary or ternary systems. In fact, there are only 12 quasicrystals of quaternaries or more in our training dataset. It is expected that stable quasicrystals will be more likely to form from systems consisting of a greater number of elements since, for instance, the number of ternary quasicrystals is much larger than the number of binary quasicrystals. On the other hand, predictions based on data science technologies are interpolative by nature, and thus it is now of particular interest to determine to what extent models trained primarily from binary and ternary systems can be generalized for multidimensional systems where less or no data are available.

With this study, we have taken the first step in the practical application of data science toward the accelerated discovery of new quasicrystals. However, there are still some technical improvements to be made. To facilitate subsequent research, we have published all datasets that were used for machine learning and benchmarking. With these datasets, all results shown in this paper can be reproduced on our platform, XenonPy. This is expected to promote comprehensive experimental validation in the quasicrystal research community.

## 4 Experimental Section

### Data Preparation

The list of 80 stable quasicrystals and 78 approximants was compiled from the Crystallography of Quasicrystals handbook. In addition, 10 090 compositions of ordinary crystals were extracted from the Materials Project database and laboratory data on failed quasicrystal syntheses. One of the difficulties in model building arose from the bias in the number of samples in different classes: 80 and 78 compositions for quasicrystals and approximants, respectively, as opposed to 126 335 crystals from the Materials Project database (V2020.08.20). Therefore, to manage the highly unbalanced class labels, the crystal data were downsampled by randomly extracting 10 000 instances from the overall data taken from the Materials Project database. After determining the hyperparameters based on the cross‐validation as described below and training the random forest classifier, the sensitivity of the prediction accuracy was examined by varying the sample size of periodic crystals from 500 to 30 000. The result has been shown in Table S4, Supporting Information. No significant change was observed in the range from 5000 to 20 000. It should also be remarked that, instead of using the Materials Project database, other databases such as ICSD,^[^ ^61^ ^]^ AFLOW,^[^ ^62^ ^]^ and NOMAD^[^ ^63^ ^]^ that provide more comprehensive lists of periodic crystals can be used.

To evaluate the validity of the predicted phase diagrams, 30 experimental phase diagrams of Al–TM–TM alloy systems were gathered from 25 papers. To facilitate the collection, an in‐house software was developed to accelerate data extraction from published phase diagram images. The difference and overlap between the extracted phase regions were quantified and quasicrystalline and approximant phase regions were predicted to evaluate the true positive and false positive rates as detailed in the Results Section.

### Compositional Pattern of Datasets

Figure 1b shows a low‐dimensional representation of the compositional distribution of the data belonging to the three classes, which was used to determine the between‐class difference and overlap. The compiled list of stable quasicrystals and approximants consisted of 26 binary, 120 ternary, and 12 quaternary compounds spanning 50 different elements. On the other hand, the ordinary crystal dataset consisted of unary to octonary systems with constituents spanning a broader range of elements. To more clearly visualize the difference and overlap in the class‐specific distributions, only binary to quaternary crystals were shown on the plot. Furthermore, crystals containing elements other than the constituents of the stable quasicrystals and approximants were excluded. Each composition was translated into a 50‐dimensional binary vector with each entry encoding the presence or absence of an element as one or zero, respectively. The feature vectors of the 19 191 compositions were projected onto a 2D subspace using a dimensionality reduction technique called UMAP.^[^ ^30^ ^]^ There was no significant bias in the distribution of the three classes at the level of their constituent elements, implying that no particular combination of elements was favorable for the formation of stable quasicrystals. The visualized data pattern also suggested that previous studies on stable quasicrystals have explored a wide range of compositional spaces without bias toward any particular compositional combination.

### Random Forest Classifier

A random forest classifier was built on an ensemble of decision tree models. The overall dataset was randomly divided into training and test sets as described in the Results Section. Cross‐validation was performed in the training dataset and the hyperparameters that minimized the prediction error were selected. The hyperparameters and search candidates are summarized in **Table** **4**. The number of combinations of search candidates was 96. As mentioned in the Results Section, the training dataset consisted of 66 stable quasicrystals and 60 approximant crystals. This dataset contained 69 unique ternary systems. In the cross‐validation, the compositional data belonging to each ternary system were lumped together, and the training and validation datasets were divided based on the ternary systems; that is, one of the 69 systems was used as the validation set, and all the remaining data, including the data outside the 69 systems, were used for training. To quantify the prediction uncertainty, models from 100 randomly selected datasets with the selected hyperparameters were also trained. Using these models, the mean and standard deviation of the performance metrics with respect to the test dataset were calculated. The learning algorithm implemented in scikit‐learn^[^ ^64^ ^]^ v0.23.1 (https://github.com/scikit‐learn/scikit‐learn/releases/tag/0.23.1) was employed to train the models.

**Table 4:** List of hyperparameters and their search candidates (grid points and module options of scikit‐learn) used for cross‐validation. The selected combination of hyperparameters is shown in bold

Hyperparameter	Search candidate
Number of trees (n_estimators)	100, **200**, 300
Maximum depth of trees (max_depth)	10, 15, 20, **25**
Number of features in each tree (max_features)	sqrt, **log**_**2**_
Bootstrap sampling in the bagging	**False**, True
Classification loss	**Entropy**, gini

## Conflict of Interest

The authors declare no conflict of interest.

## Author Contributions

R.Y. and K.K. designed the conceptual idea and proof outline. C.L. and R.Y. wrote the manuscript and carried out the data analysis. E.F., Y.K., Y.I., A.I., R.T., and K.K. worked out the collection and curation of the dataset. All authors discussed the results and commented on the manuscript.

## Supporting information

Supporting Information

Supporting Information

## Data Availability

The data that support the findings of this study are available from the corresponding author upon reasonable request.
