# Supplementary material for: Machine Learning to Predict Quasicrystals from Chemical Compositions
Source: Adv Mater. 2021 Jul 19;33(36):2102507. doi: 10.1002/adma.202102507 (PMC11468293; doi:10.1002/adma.202102507)
Supplement: Supplementary file 1 — Supporting Information [file ADMA-33-2102507-s001.pdf]

© 2021 Wiley-VCH GmbH

ADVANCED MATERIALS

Supporting Information

for *Adv. Mater.,* DOI: 10.1002/adma.202102507

Machine Learning to Predict Quasicrystals from Chemical Compositions

Chang Liu, Erina Fujita, Yukari Katsura, Yuki Inada, Asuka Ishikawa, Ryuji Tamura, Kaoru Kimura, and Ryo Yoshida**

Supplementary Note

Machine learning to predict quasicrystals from chemical compositions

Chang Liu Erina Fujita Yukari Katsura Yuki Inada Asuka Ishikawa Ryuji Tamura Kaoru Kimura Ryo Yoshida**

C. Liu, R. Yoshida
Research Organization of Information and Systems, The Institute of Statistical Mathematics, Tachikawa, 190-8562, Japan
E. Fujita, Y. Katsura, Y. Inada, K. Kimura
The University of Tokyo, Department of Advanced Materials Science, Kashiwa, 277-8561, Japan
A. Ishikawa, R. Tamura
Tokyo University of Science, Department of Materials Science and Technology, Tokyo, 125–8585, Japan
R. Yoshida
National Institute for Materials Science, Research and Service Division of Materials Data and Integrated System, Tsukuba, 305-0047, Japan
R. Yoshida
The Graduate University for Advanced Studies, Department of Statistical Science, Tachikawa, 190-8562, Japan
R. Yoshida
Email Address: yoshidar@ism.ac.jp
K. Kimura
Email Address: bkimura@phys.mm.t.u-tokyo.ac.jp

Quasicrystals and approximants used in the supervised learning

Table S1: List of quasicrystals and approximants that were used in the supervised learning. Each column gives the chemical composition, the label indicating quasicrystal or approximant (QC and AC), structure type, the known space group, and the guessed space group, respectively. The data were collected from the Crystallography of Quasicrystals handbook [1].

Composition	QC or AP	Type	Space group	Guessed space group
**Cr 5 Ni 3 Si 2**	QC	Octagonal		I8_4/mcm
**Mn 4 Si 1**	QC	Octagonal		I8_4/mcm
**Mn 82 Si 15 Al 3**	QC	Octagonal	I8_4/mcm	I8_4/mcm
**Zn 58 Mg 40 Dy 2**	QC	Decagonal		P10_5/mmc
**Zn 58 Mg 40 Dy 2**	QC	Decagonal		P10_5/mmc
**Zn 58 Mg 40 Er 2**	QC	Decagonal		P10_5/mmc
**Zn 58 Mg 40 Ho 2**	QC	Decagonal		P10_5/mmc
**Zn 58 Mg 40 Lu 2**	QC	Decagonal		P10_5/mmc
**Zn 58 Mg 40 Tm 2**	QC	Decagonal		P10_5/mmc
**Zn 58 Mg 40 Y 2**	QC	Decagonal		P10_5/mmc
**Al 70 Ni 15 Fe 15**	QC	Decagonal		P10_5/mmc
**Al 65 Cu 15 Rh 20**	QC	Decagonal		P10_5/mmc
**Al 70 Ni 20 Rh 10**	QC	Decagonal		P10_5/mmc
**Al 65 Cu 20 Co 15**	QC	Decagonal		P10_5/mmc
**Al 65 Cu 20 Ir 15**	QC	Decagonal		P10_5/mmc
**Al 73.2 Co 26.8**	AP	Decagonal	P2	P2
**Al 71.5 Co 28.5**	AP	Decagonal	P6_3/mmc	P6_3/mmc
**Al 71.5 Co 23.6 Cu 4.9**	AP	Decagonal	Cm	Cm
**Al 76 Co 24**	AP	Decagonal	Pmn2_1	Pmn2_1
**Al 74.8 Co 17.0 Ni 8.2**	AP	Decagonal	Immm	Immm
**Al 71.8 Co 21.1 Ni 7.1**	AP	Decagonal	Cm	Cm
**Al 70.5 Mn 16.5 Pd 13**	QC	Decagonal	P10_5/mmc	P10_5/mmc
**Al 40 Mn 25 Fe 15 Ge 20**	QC	Decagonal		P10_5/mmc
**Ga 33 Fe 46 Cu 3 Si 18**	QC	Decagonal		P10_5/mmc
**Ga 43 Co 47 Cu 10**	QC	Decagonal		P10_5/mmc
**Ga 35 V 45 Ni 6 Si 14**	QC	Decagonal		P10_5/mmc
**Al 75 Mn 20 Pd 5**	AP	Decagonal	Pnma	Pnma
**Al 75 Os 10 Pd 15**	QC	Decagonal	P10_5/mmc	P10_5/mmc
**Al 75 Ru 10 Pd 15**	QC	Decagonal		P10_5/mmc
**Al 73 Ir 14.5 Os 12.5**	QC	Decagonal	P10_c	P10_c
**Al 70 Ni 20 Ru 10**	QC	Decagonal	P10_5/mmc	P10_5/mmc
**Al 72.5 Pd 27.5**	AP 2/1, 1/1	Decagonal	Pna2_1	Pna2_1
**Al 75 Pd 13 Ru 12**	AP 3/2, 2/1	Decagonal	Pna2_1	Pna2_1
**Ni 70.6 Cr 29.4**	QC	Dodecagonal		P(-12)m2
**V 3 Ni 2**	QC	Dodecagonal		P(-12)m2
**V 15 Ni 10 Si 1**	QC	Dodecagonal		P(-12)m2
**Ta 1.6 Te 1**	QC	Dodecagonal	P(-12)m2	P(-12)m2
**Ta 97 Te 60**	AP	Dodecagonal	P2_12_12_1	P2_12_12_1
**Ta 181 Te 112**	AP	Dodecagonal	P2_12_12_1	P2_12_12_1
**Al 65 Cu 20 Ru 15**	QC	Icosahedral	Fm-3-5	Fm-3-5
**Al 65 Cu 20 Os 15**	QC	Icosahedral	Fm-3-5	Fm-3-5
**Al 65 Cu 20 Fe 15**	QC	Icosahedral	Fm-3-5	Fm-3-5

Table S1 continued

Composition	QC or AP	Type	Space group	Guessed space group
**Al 70.5 Pd 21 Mn 8.5**	QC	Icosahedral	Fm-3-5	Fm-3-5
**Al 70 Pd 20 Re 10**	QC	Icosahedral	Fm-3-5	Fm-3-5
**Al 70 Pd 21 Tc 9**	QC	Icosahedral	Fm-3-5	Fm-3-5
**Al 71 Pd 21 Re 8**	QC	Icosahedral	Pm-3-5	Pm-3-5
**Al 72 Pd 17 Ru 11**	QC	Icosahedral	Fm-3-5	Fm-3-5
**Al 72 Pd 17 Os 11**	QC	Icosahedral	Fm-3-5	Fm-3-5
**Al 71.5 Cu 8.5 Ru 20**	AP 1/0	Icosahedral	P2_13	P2_13
**Al 55.1 Cu 14.6 Ru 20.2 Si 10.1**	AP 1/0×2 (γ)	Icosahedral	Fm-3	Fm-3
**Al 39 Pd 21 Fe 2**	AP 1/0×2 (c)	Icosahedral	Fm-3	Fm-3
**Al 68 Pd 20 Ru 12**	AP 1/0×2 (c)	Icosahedral	P23	P23
**Al 57.3 Cu 31.4 Ru 11.3**	AP 1/1	Icosahedral	Pm-3	Pm-3
**Al 55 Cu 25.5 Fe 12.5 Si 7**	AP 1/1	Icosahedral	Pm-3	Pm-3
**Al 40 Mn 10.1 Si 7.4**	AP 1/1	Icosahedral	Pm-3	Pm-3
**Al 67 Pd 11 Mn 14 Si 7**	AP 1/1	Icosahedral	Pm-3	Pm-3
**Al 66.6 Rh 26.1 Si 7.3**	AP 2/1	Icosahedral	Pm-3	Pm-3
**Al 70 Pd 23 Mn 6 Si 1**	AP 2/1	Icosahedral	Pm-3	Pm-3
**Zn 76 Mg 17 Hf 7**	QC	Icosahedral	Fm-3-5	Fm-3-5
**Zn 84 Mg 7 Zr 9**	QC	Icosahedral	Pm-3-5	Pm-3-5
**Al 6 Cu 1 Li 3**	QC	Icosahedral	Pm-3-5	Pm-3-5
**Mg 43 Al 42 Pd 15**	QC	Icosahedral	Pm-3-5	Pm-3-5
**Zn 40 Mg 39.5 Ga 25**	QC	Icosahedral	Pm-3-5	Pm-3-5
**Zn 74 Mg 15 Ho 11**	QC	Icosahedral	Pm-3-5	Pm-3-5
**Zn 56.8 Mg 34.6 Tb 8.7**	QC	Icosahedral	Fm-3-5	Fm-3-5
**Zn 65 Mg 26 Ho 9**	QC	Icosahedral	Fm-3-5	Fm-3-5
**Zn 64 Mg 25 Y 11**	QC	Icosahedral	Fm-3-5	Fm-3-5
**Zn 55 Mg 40 Nd 5**	QC	Icosahedral	Pm-3-5	Pm-3-5
**Zn 56.8 Mg 34.6 Dy 8.7**	QC	Icosahedral	Fm-3-5	Fm-3-5
**Zn 77 Mg 17.5 Ti 5.5**	AP 1/1	Icosahedral	Pm-3	Pm-3
**Zn 77 Mg 18 Hf 5**	AP 1/1	Icosahedral	Pm-3	Pm-3
**Zn 77 Mg 18 Zr 5**	AP 1/1	Icosahedral	Pm-3	Pm-3
**Al 88.6 Cu 19.4 Li 50.3**	AP 1/1	Icosahedral	Im-3	Im-3
**Zn 34.6 Mg 40 Al 25.4**	AP 1/1	Icosahedral	Im-3	Im-3
**Zn 61.4 Mg 24.5 Er 14.1**	AP 2/1	Icosahedral	F-43m	F-43m
**Zn 37 Mg 46 Al 17**	AP 2/1	Icosahedral	Pm-3	Pm-3
**Zn 47.3 Mg 27 Al 10.7**	AP 2/1	Icosahedral	Pa-3	Pa-3
**Zn 73.6 Mg 2.5 Sc 11.2**	AP 2/1	Icosahedral	Pa-3	Pa-3
**Zn 40 Mg 39.5 Ga 16.4 Al 4.1**	AP 3/2-2/1-2/1	Icosahedral	Cmc2_1	Cmc2_1
**Cu 48 Sc 15 Ga 34 Mg 3**	QC	Icosahedral		Pm-3-5
**Cu 46 Sc 16 Al 38**	QC	Icosahedral		Pm-3-5
**Zn 84 Ti 8 Mg 8**	QC	Icosahedral		Pm-3-5
**Zn 75 Sc 15 Ni 10**	QC	Icosahedral		Pm-3-5
**Zn 72 Sc 16 Cu 12**	QC	Icosahedral		Pm-3-5
**Zn 75 Sc 15 Co 10**	QC	Icosahedral		Pm-3-5
**Zn 75 Sc 15 Fe 10**	QC	Icosahedral		Pm-3-5
**Zn 75 Sc 15 Mn 10**	QC	Icosahedral		Pm-3-5
**Zn 80 Sc 15 Mg 5**	QC	Icosahedral	Pm-3-5	Pm-3-5
**Zn 75 Sc 15 Pt 10**	QC	Icosahedral		Pm-3-5
**Zn 75 Sc 15 Pd 10**	QC	Icosahedral		Pm-3-5

Table S1 continued

Composition	QC or AP	Type	Space group	Guessed space group
**Zn 75 Sc 15 Au 10**	QC	Icosahedral		Pm-3-5
**Zn 75 Sc 15 Ag 10**	QC	Icosahedral		Pm-3-5
**Zn 77 Sc 8 Ho 8 Fe 7**	QC	Icosahedral		Pm-3-5
**Zn 77 Sc 8 Er 8 Fe 7**	QC	Icosahedral		Pm-3-5
**Zn 77 Sc 7 Tm 9 Fe 7**	QC	Icosahedral		Pm-3-5
**Zn 56.8 Er 8.7 Mg 34.6**	QC	Icosahedral	Fm-3-5	Fm-3-5
**Zn 76 Yb 14 Mg 10**	QC	Icosahedral		Fm-3-5
**Cd 65 Mg 20 Lu 15**	QC	Icosahedral		Fm-3-5
**Ag 42 In 42 Yb 16**	QC	Icosahedral	Pm-3-5	Pm-3-5
**Cd 65 Mg 20 Tm 15**	QC	Icosahedral		Pm-3-5
**Ag 42 In 42 Ca 16**	QC	Icosahedral	Pm-3-5	Pm-3-5
**Au 44.2 In 41.7 Ca 14.1**	QC	Icosahedral	Pm-3-5	Pm-3-5
**Cd 65 Mg 20 Y 15**	QC	Icosahedral	Pm-3-5	Pm-3-5
**Cd 65 Mg 20 Er 15**	QC	Icosahedral	Fm-3-5	Fm-3-5
**Cd 65 Mg 20 Ho 15**	QC	Icosahedral	Fm-3-5	Fm-3-5
**Cd 65 Mg 20 Tb 15**	QC	Icosahedral	Fm-3-5	Fm-3-5
**Cd 65 Mg 20 Dy 15**	QC	Icosahedral	Fm-3-5	Fm-3-5
**Cd 65 Mg 20 Gd 15**	QC	Icosahedral		Fm-3-5
**Cd 84 Yb 16**	QC	Icosahedral	Pm-3-5	Pm-3-5
**Cd 65 Mg 20 Yb 15**	QC	Icosahedral		Pm-3-5
**Cd 85 Ca 15**	QC	Icosahedral		Pm-3-5
**Cd 65 Mg 20 Ca 15**	QC	Icosahedral	Pm-3-5	Pm-3-5
**Be 17 Ru 3**	AP 1/1	Icosahedral		Im-3
**Ga 3.85 Ni 2.15 Hf 1**	AP 1/1	Icosahedral		Im-3
**Ga 3.22 Ni 2.78 Zr 1**	AP 1/1	Icosahedral		Im-3
**Ga 3.64 Ni 2.36 Sc 1**	AP 1/1	Icosahedral		Im-3
**Ga 2.3 Cu 3.7 Sc 1**	AP 1/1	Icosahedral		Im-3
**Ga 2.6 Cu 3.4 Lu 1**	AP 1/1	Icosahedral		Im-3
**Zn 17 Sc 3**	AP 1/1	Icosahedral		Im-3
**Zn 17 Yb 3**	AP 1/1	Icosahedral		Im-3
**Ag 42.5 Ga 42.5 Yb 15**	AP 1/1	Icosahedral		Im-3
**Au 50.5 Ga 35.9 Ca 13.6**	AP 1/1	Icosahedral	Im-3	Im-3
**Au 61.2 Sn 23.9 Dy 15.2**	AP 1/1	Icosahedral		Im-3
**Au 62.3 Sn 23.1 Gd 14.6**	AP 1/1	Icosahedral		Im-3
**Ag 42.2 In 42.6 Tm 15.2**	AP 1/1	Icosahedral		Im-3
**Au 47.2 In 37.2 Gd 15.6**	AP 1/1	Icosahedral		Im-3
**Au 12.2 In 6.3 Ca 3**	AP 1/1	Icosahedral	Im-3	Im-3
**Au 64.2 Sn 21.3 Pr 14.5**	AP 1/1	Icosahedral		Im-3
**Au 65 Sn 20 Ce 15**	AP 1/1	Icosahedral	Im-3	Im-3
**Ag 46.4 In 39.7 Gd 13.9**	AP 1/1	Icosahedral		Im-3
**Au 49.7 In 35.4 Ce 14.9**	AP 1/1	Icosahedral		Im-3
**Au 60.7 Sn 25.2 Eu 14.1**	AP 1/1	Icosahedral		Im-3
**Ag 2 In 4 Yb 1**	AP 1/1	Icosahedral	Im-3	Im-3
**Ag 46.9 In 38.7 Pr 14.4**	AP 1/1	Icosahedral		Im-3
**Au 42 In 42 Yb 16**	AP 1/1	Icosahedral	Ia-3	Ia-3
**Cd 6 Gd 1**	AP 1/1	Icosahedral	Im-3	Im-3
**Ag 2 In 4 Ca 1**	AP 1/1	Icosahedral	Im-3	Im-3
**Ag 47.7 In 38.7 Ce 14.2**	AP 1/1	Icosahedral		Im-3

Table S1 continued

Composition	QC or AP	Type	Space group	Guessed space group
**Cd 6 Dy 1**	AP 1/1	Icosahedral	Im-3	Im-3
**Cd 6 Y 1**	AP 1/1	Icosahedral		Im-3
**Cd 6 Sm 1**	AP 1/1	Icosahedral	Im-3	Im-3
**Cd 6 Nd 1**	AP 1/1	Icosahedral	Im-3	Im-3
**Cd 6 Yb 1**	AP 1/1	Icosahedral	Im-3	Im-3
**Ag 42.9 In 43.6 Eu 13.5**	AP 1/1	Icosahedral		Im-3
**Cd 6 Ca 1**	AP 1/1	Icosahedral	Im-3	Im-3
**Cd 25 Eu 4**	AP 1/1×2	Icosahedral	Fd-3	Fd-3
**Cd 19 Pr 3**	AP 1/1	Icosahedral	Im-3	Im-3
**Cd 6 Sr 1**	AP 1/1	Icosahedral		Im-3
**Au 61.1 Ga 25.0 Ca 13.9**	AP 2/1	Icosahedral	Pa-3	Pa-3
**Au 60.3 Sn 24.6 Yb 15.1**	AP 2/1	Icosahedral		Pa-3
**Au 61.2 Sn 24.3 Ca 14.5**	AP 2/1	Icosahedral		Pa-3
**Au 42.9 In 41.9 Yb 15.2**	AP 2/1	Icosahedral	Pa-3	Pa-3
**Au 37 In 39.6 Ca 12.6**	AP 2/1	Icosahedral	Pa-3	Pa-3
**Ag 41.7 In 43.2 Yb 15.1**	AP 2/1	Icosahedral	Pa-3	Pa-3
**Au 61.2 Sn 24.5 Eu 14.3**	AP 2/1	Icosahedral		Pa-3
**Ag 41 In 44 Yb 15**	AP 2/1	Icosahedral		Pa-3
**Ag 42 In 45 Ca 13**	AP 2/1	Icosahedral	Pa-3	Pa-3
**Cd 76 Ca 13**	AP 2/1	Icosahedral	Pa-3	Pa-3
**Ag 43.4 In 42.8 Eu 13.8**	AP 2/1	Icosahedral	Pa-3	Pa-3

Element features

Table S2: 58 element features that were used to calculate the compositional descriptors. The dataset is accessible at our open-source software XenonPy [2].

Feature ID	Description	Unit	Reference
**atomic_number**	Atomic number		
**atomic_radius**	Atomic radius	pm	[3, 4]
**atomic_radius_rahm**	Atomic radius by Rahm et al.	pm	[4–6]
**atomic_volume**	Atomic volume	$cm^3\ mol^{-1}$	[4]
**atomic_weight**	Atomic weight		[4, 7, 8]
**boiling_point**	Boiling temperature	K	[4]
**bulk_modulus**	Bulk modulus	GPa	[9]
**c6_gb**	C_6 dispersion coefficient in a.u. (Gould & Bučko)	a.u.	[4, 10, 11]
**covalent_radius_cordero**	Covalent radius by Cerdero et al.	pm	[4, 12]
**covalent_radius_pyykko**	Single bond covalent radius by Pyykko et al.	pm	[4, 13]
**covalent_radius_pyykko_double**	Double bond covalent radius by Pyykko et al.	pm	[4, 14]
**covalent_radius_pyykko_triple**	Triple bond covalent radius by Pyykko et al.	pm	[4, 15]
**covalent_radius_slater**	Covalent radius by Slater	pm	[3]
**density**	Density at 295K	$g\ cm^3$	[4]
**dipole_polarizability**	Dipole polarizability	a.u,	[4, 16]
**electron_negativity**	Pauling electronegativity		[17]
**electron_affinity**	Electron affinity	eV	[4, 18, 19]
**en_allen**	Allen's scale of electronegativity	eV	[4, 20, 21]
**en_ghosh**	Ghosh's scale of electronegativity		[4, 22]
**en_pauling**	Pauling's scale of electronegativity		[4, 18]
**first_ion_en**	First ionization energy	eV	[18]
**fusion_enthalpy**	Enthalpy of fusion for elements at their melting temperatures	$kJ\ mol^{-1}$	[18]
**gs_bandgap**	DFT bandgap energy of T=0K ground state	eV	[23, 24]
**gs_energy**	DFT energy per atom (raw VASP value) of T=0K ground state	$eV\ atom^{-1}$	[23, 24]
**gs_est_bcc_latcnt**	Estimated BCC lattice parameter based on the DFT volume of the OQMD ground state for each element		[23, 24]
**gs_est_fcc_latcnt**	Estimated FCC lattice parameter based on the DFT volume of the OQMD ground state for each element	Å	[23, 24]
**gs_mag_moment**	DFT magnetic moment of T=0K ground state		[23, 24]
**gs_volume_per**	DFT volume per atom of T=0K ground state	$Å^3\ atom^{-1}$	[23, 24]
**hhi_p**	HerfindahlHirschman Index (HHI) production values		[25]
**hhi_r**	HerfindahlHirschman Index (HHI) reserves values		[25]

Table S2 continued

Feature ID	Description	Unit	Reference
**heat_capacity_mass**	Specific heat capacity at STP[1]	J mol^{-1} K^{-1}	[18]
**heat_capacity_molar**	Molar heat capacity at STP	J mol^{-1} K^{-1}	[18]
**icsd_volume**	Volume per atom of ICSD phae at STP		[26–28]
**evaporation_heat**	Evaporation heat	kJ mol^{-1}	[4]
**heat_of_formation**	Heat of formation	kJ mol^{-1}	[4]
**lattice_constant**	Lattice constant	Å	[4]
**mendeleev_number**	Mendeleev's number		[4, 29, 30]
**melting_point**	Melting temperature	K	[4]
**molar_volume**	Molar volume	L mol^{-1}	[9]
**num_unfilled**	Number of unfilled valence orbitals		[31, 32]
**num_valance**	Number of valence electrons		[31, 32]
**num_d_unfilled**	Number of unfilled d valence orbitals		[31, 32]
**num_d_valence**	Number of filled d valence orbitals		[31, 32]
**num_f_unfilled**	Number of unfilled f valence orbitals		[31, 32]
**num_f_valence**	Number of filled f valence orbitals		[31, 32]
**num_p_unfilled**	Number of unfilled p valence orbitals		[31, 32]
**num_p_valence**	Number of filled p valence orbitals		[31, 32]
**num_s_unfilled**	Number of unfilled s valence orbitals		[31, 32]
**num_s_valence**	Number of filled s valence orbitals		[31, 32]
**period**	Period in periodic table		[4]
**specific_heat**	Specific heat at 20 °C	J g^{-1} mol^{-1}	[4]
**thermal_conductivity**	Thermal conductivity at 25 °C	W m^{-1} K^{-1}	[4]
**vdw_radius**	van der Waals radius	pm	[4, 18]
**vdw_radius_alvarez**	van der Waals radius according to Alvarez	pm	[4, 33]
**vdw_radius_mm3**	van der Waals radius from the MM3 FF	pm	[4, 34]
**vdw_radius_uff**	van der Waals radius from the UFF	pm	[4, 35]
**sound_velocity**	Velocity of sound	m s^{-1}	[9]
**polarizability**	Static average electric dipole polarizability	$10^{-24}cm^3$	[18]

[1] Standard Temperature and Pressure

Generalization ability of the model

Table S3: Prediction performance for the three-class classification problem of stable quasicrystals (QC), approximants (AC), and others. The left table is the confusion matrix, and the right table reports the per-class recall, precision, and F_1 metrics. The performance metrics were averaged over 100 different bootstrap sets, and the numbers in parentheses represent the standard deviations. The results labeled as "with Al systems" are the same as we report in Table 1. Additionally, in the "without Al systems" panel, we show the results of the performance evaluation after eliminating the aluminum-containing compositions from the test instances.

			Predicted class		
	Test set		QC	AC	others
True class	With Al systems	QC	9.63 (1.641)	3.24 (1.342)	3.13 (1.189)
		AC	3.11 (1.555)	9.73 (1.805)	3.16 (1.573)
		others	0.76 (0.896)	0.42 (0.619)	2016.82 (1.024)
	Without Al systems	QC	7.14 (1.594)	1.91 (1.150)	1.90 (1.221)
		AC	1.43 (1.185)	7.68 (1.788)	2.36 (1.389)
		others	0.45 (0.698)	0.16 (0.367)	1900.13 (8.795)

	Performance metrics		
	Recall	Precision	F_1
QC	0.602 (0.103)	0.722 (0.090)	0.650 (0.076)
AC	0.608 (0.113)	0.731 (0.089)	0.658 (0.088)
others	0.999 (0.001)	0.997 (0.001)	0.998 (0.001)
QC	0.662 (0.140)	0.807 (0.125)	0.715 (0.102)
AC	0.671 (0.137)	0.792 (0.110)	0.720 (0.108)
others	1.000 (0.000)	0.998 (0.001)	0.999 (0.001)

Prediction accuracy sensitivity

Table S4: The sensitivity of the prediction accuracy when using the different sample sizes of periodic crystals ($N_{crystal}$). The left table is the confusion matrix, and the right table reports the per-class recall, precision, and F_1 metrics. The performance metrics were averaged over 100 different bootstrap sets, and the numbers in parentheses represent the standard deviations.

		Predicted class		
$N_{crystal}$ (True class)		QC	AC	others
500	QC	11.08 (2.58)	3.58 (1.97)	0.88 (1.04)
	AC	3.48 (1.75)	10.75 (2.98)	1.13 (1.12)
	others	0.61 (0.84)	0.35 (0.64)	118.14 (4.25)
2,500	QC	9.90 (3.10)	3.65 (1.80)	2.19 (1.44)
	AC	3.48 (1.82)	9.88 (2.59)	2.03 (1.38)
	others	0.38 (0.61)	0.49 (0.67)	518.00 (4.82)
5,000	QC	9.96 (2.83)	3.47 (1.77)	2.40 (1.41)
	AC	2.95 (1.81)	9.67 (2.75)	2.84 (1.68)
	others	0.37 (0.67)	0.66 (0.78)	1017.68 (4.86)
10,000	QC	9.73 (2.75)	3.17 (1.62)	3.02 (1.68)
	AC	2.68 (1.70)	8.54 (2.33)	3.67 (1.98)
	others	0.50 (0.70)	0.72 (0.83)	2017.97 (5.19)
15,000	QC	9.47 (2.94)	3.33 (2.04)	3.49 (1.58)
	AC	2.90 (1.66)	8.46 (2.22)	4.65 (2.44)
	others	0.49 (0.69)	1.18 (1.03)	3016.03 (4.84)
20,000	QC	9.39 (2.69)	2.85 (1.89)	3.92 (2.12)
	AC	2.74 (1.68)	8.80 (2.68)	4.78 (2.53)
	others	0.64 (0.83)	1.26 (1.08)	4015.62 (5.11)
25,000	QC	9.29 (2.68)	2.64 (1.57)	3.83 (1.77)
	AC	2.84 (1.77)	7.70 (2.27)	5.75 (2.51)
	others	0.71 (0.85)	1.16 (1.08)	5016.08 (4.96)
30,000	QC	9.45 (2.77)	2.65 (1.61)	4.20 (1.83)
	AC	2.32 (1.39)	7.37 (2.30)	5.66 (2.47)
	others	0.83 (0.93)	1.45 (1.19)	6016.07 (4.79)

	Performance metrics		
	Recall	Precision	F_1
QC	0.719 (0.121)	0.736 (0.107)	0.719 (0.086)
AC	0.701 (0.125)	0.735 (0.127)	0.708 (0.096)
others	0.992 (0.009)	0.983 (0.012)	0.988 (0.007)
QC	0.627 (0.132)	0.718 (0.120)	0.661 (0.109)
AC	0.645 (0.120)	0.708 (0.107)	0.668 (0.093)
others	0.998 (0.002)	0.992 (0.004)	0.995 (0.002)
QC	0.627 (0.123)	0.756 (0.128)	0.677 (0.102)
AC	0.631 (0.124)	0.704 (0.120)	0.655 (0.096)
others	0.999 (0.001)	0.995 (0.002)	0.997 (0.001)
QC	0.609 (0.135)	0.758 (0.127)	0.666 (0.112)
AC	0.584 (0.127)	0.694 (0.112)	0.623 (0.091)
others	0.999 (0.001)	0.997 (0.001)	0.998 (0.001)
QC	0.582 (0.133)	0.736 (0.113)	0.641 (0.105)
AC	0.536 (0.123)	0.663 (0.127)	0.581 (0.098)
others	0.999 (0.000)	0.997 (0.001)	0.998 (0.001)
QC	0.585 (0.128)	0.739 (0.118)	0.643 (0.101)
AC	0.548 (0.133)	0.688 (0.136)	0.597 (0.107)
others	1.000 (0.000)	0.998 (0.001)	0.999 (0.000)
QC	0.593 (0.122)	0.728 (0.138)	0.644 (0.107)
AC	0.479 (0.129)	0.677 (0.133)	0.550 (0.116)
others	1.000 (0.000)	0.998 (0.001)	0.999 (0.000)
QC	0.583 (0.123)	0.750 (0.117)	0.648 (0.102)
AC	0.483 (0.122)	0.642 (0.145)	0.542 (0.113)
others	1.000 (0.000)	0.998 (0.000)	0.999 (0.000)

Phase diagram

Figure S1: Predicted and experimentally determined phase diagrams for the 30 Al-TM-TM alloy systems. For each system, the left panel shows the predicted diagram made by the random forest classifier, and the right panel shows the actual phase diagram extracted from the literature. Three different colors are used to distinguish the phase region of quasicrystals, approximants, and others.

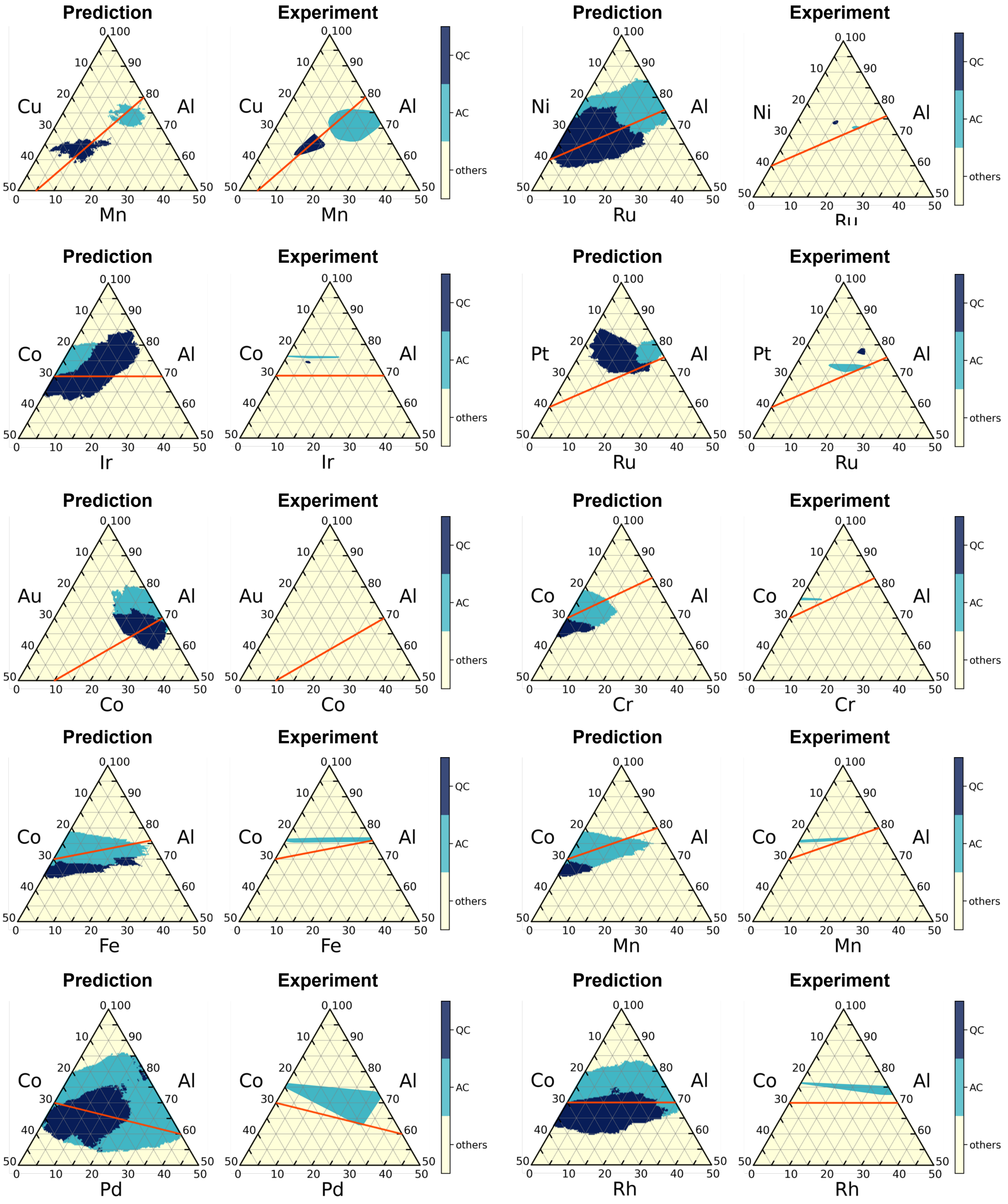

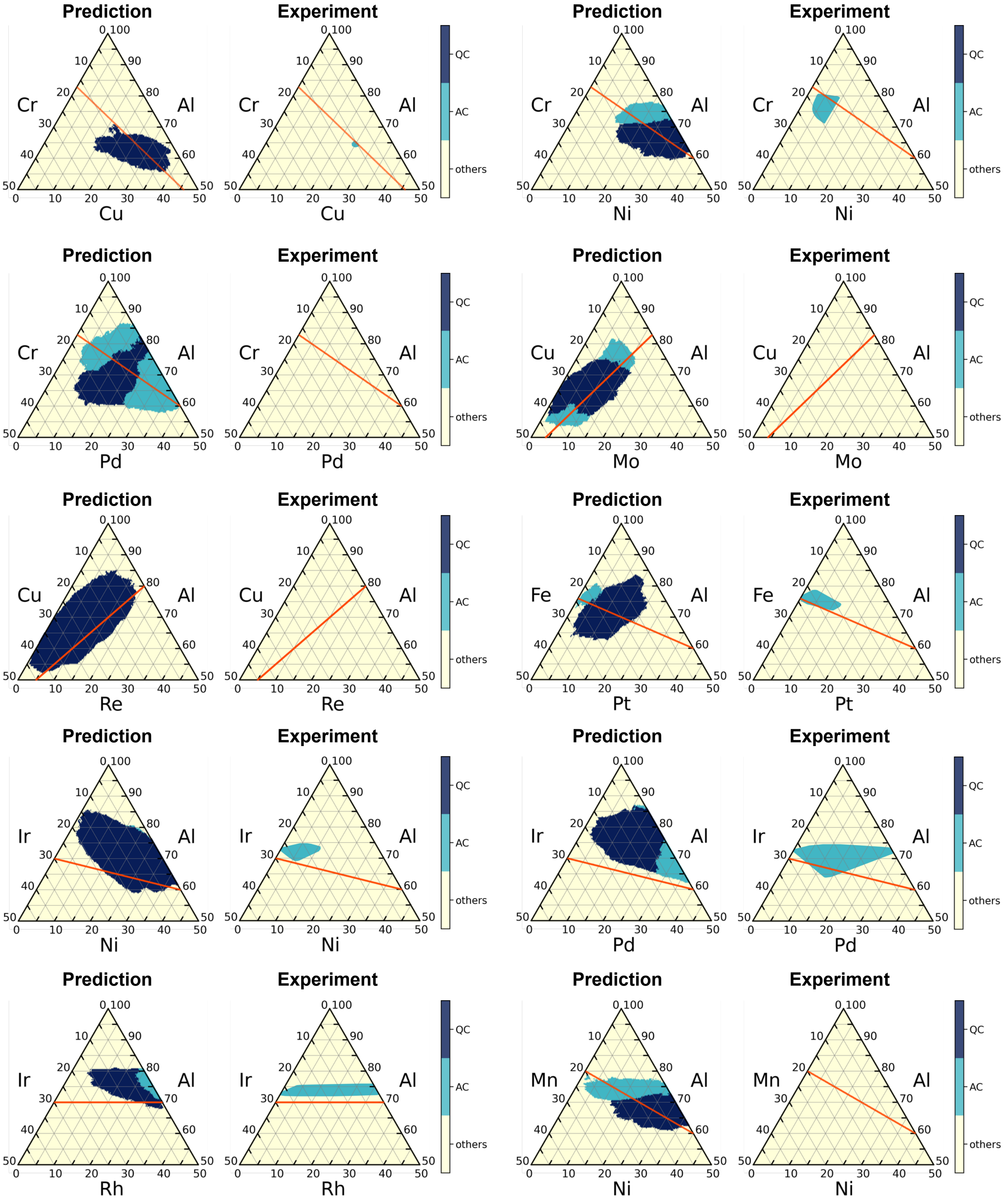

Prediction
Experiment
Cr
Al
Cu
QC
AC
others
Prediction
Experiment
Cr
Al
Ni
QC
AC
others
Prediction
Experiment
Cr
Al
Pd
QC
AC
others
Prediction
Experiment
Cu
Al
Mo
QC
AC
others
Prediction
Experiment
Cu
Al
Re
QC
AC
others
Prediction
Experiment
Fe
Al
Pt
QC
AC
others
Prediction
Experiment
Ir
Al
Ni
QC
AC
others
Prediction
Experiment
Ir
Al
Pd
QC
AC
others
Prediction
Experiment
Ir
Al
Rh
QC
AC
others
Prediction
Experiment
Mn
Al
Ni
QC
AC
others
0 100
10 90
20 80
30 70
40 60
50 50
0 10 20 30 40 50

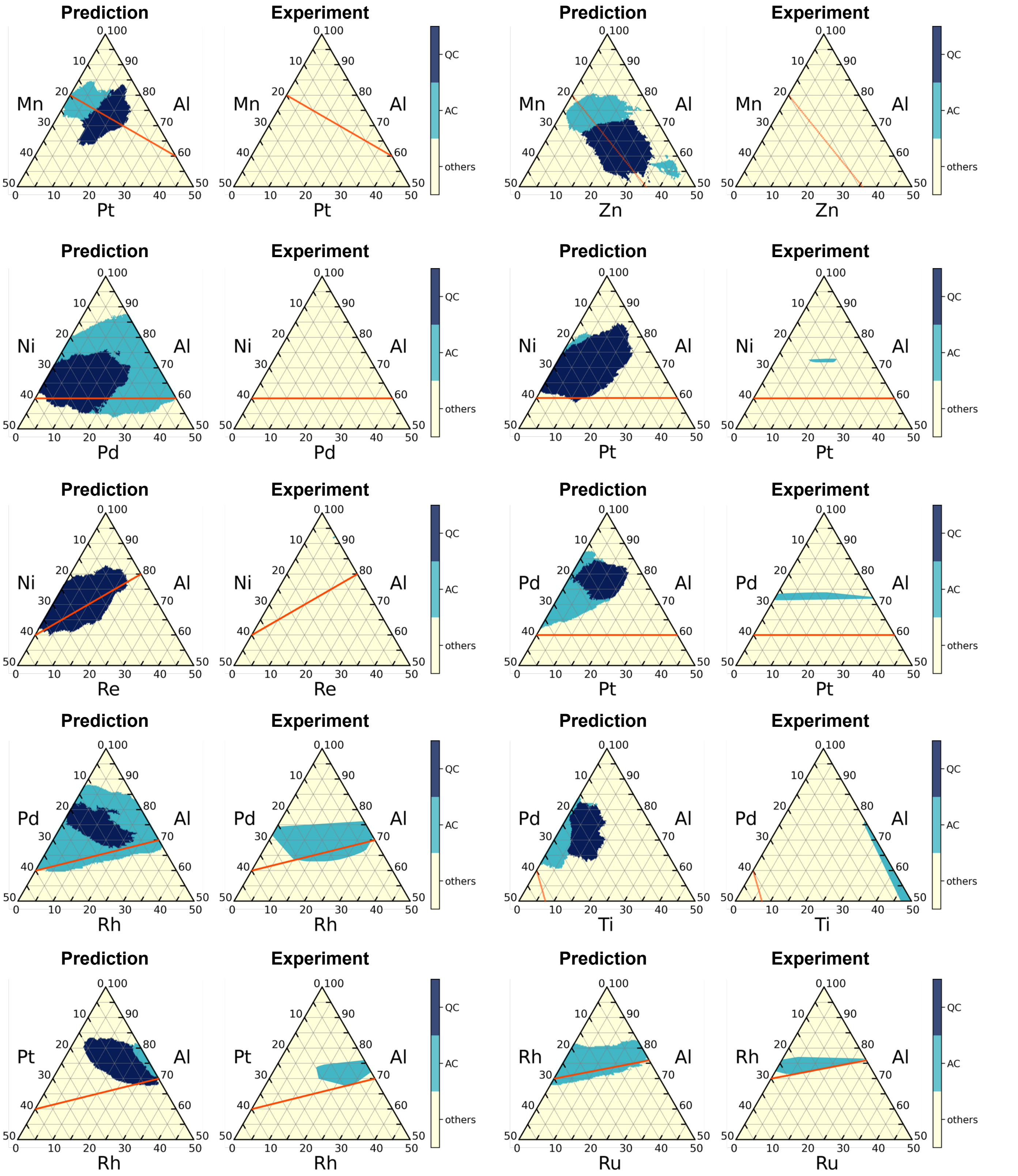
Prediction
Experiment
Prediction
Experiment
Mn
Al
Pt
Mn
Al
Pt
QC
AC
others
Mn
Al
Zn
Mn
Al
Zn
QC
AC
others
Prediction
Experiment
Prediction
Experiment
Ni
Al
Pd
Ni
Al
Pd
QC
AC
others
Ni
Al
Pt
Ni
Al
Pt
QC
AC
others
Prediction
Experiment
Prediction
Experiment
Ni
Al
Re
Ni
Al
Re
QC
AC
others
Pd
Al
Pt
Pd
Al
Pt
QC
AC
others
Prediction
Experiment
Prediction
Experiment
Pd
Al
Rh
Pd
Al
Rh
QC
AC
others
Pd
Al
Ti
Pd
Al
Ti
QC
AC
others
Prediction
Experiment
Prediction
Experiment
Pt
Al
Rh
Pt
Al
Rh
QC
AC
others
Rh
Al
Ru
Rh
Al
Ru
QC
AC
others

Machine learning learned rules

Figure S2: The formation rules of QC/AC discovered by the machine learning algorithm are overwritten on the predicted phase diagrams of the 30 Al-TM-TM systems. A color-coded line represents a condition imposed on the weighted average of van der Waals radius, electronegativity, first ionisation energy, the number of filled p valence orbitals, or the energy per atom at T=0K ground state.

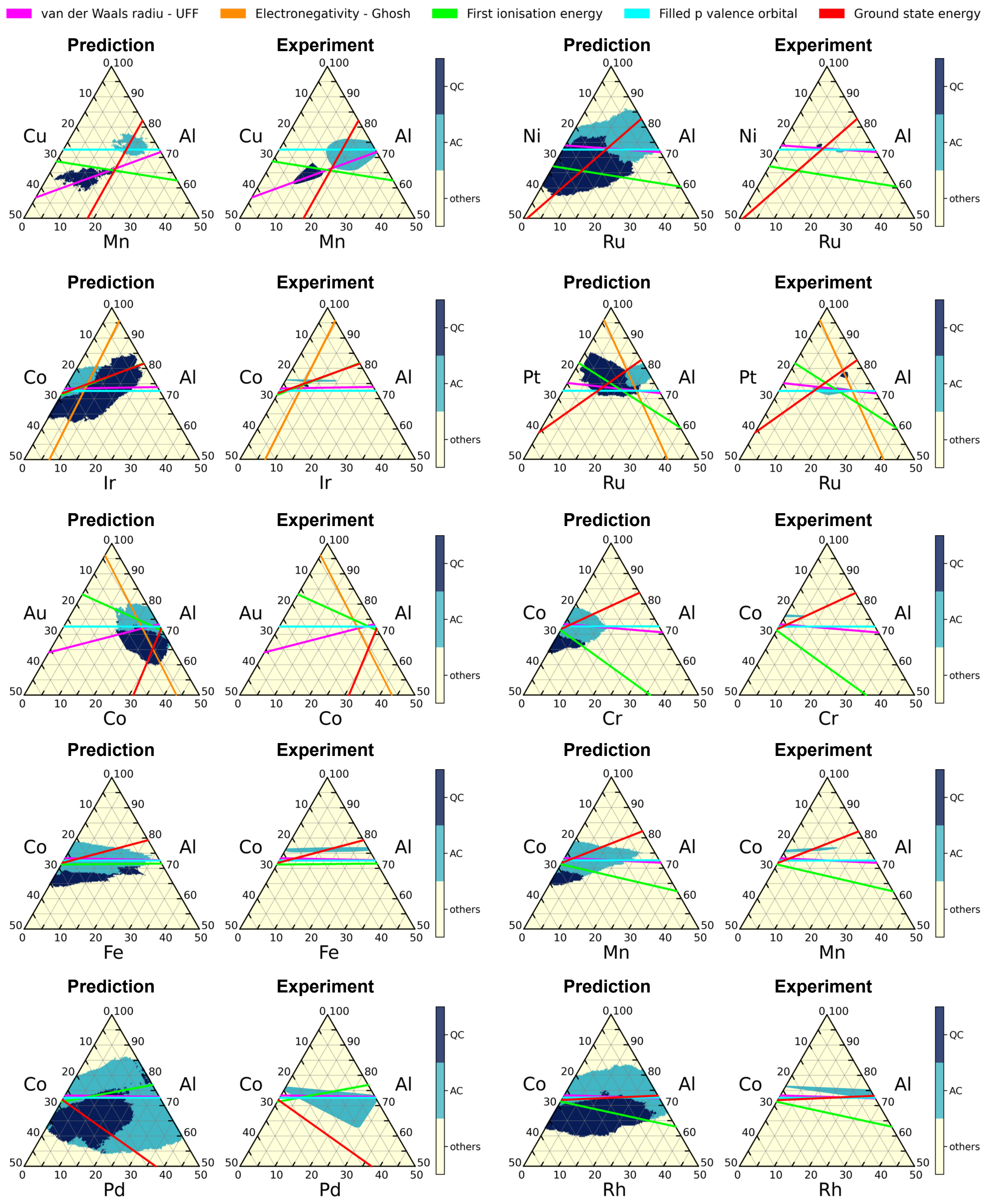

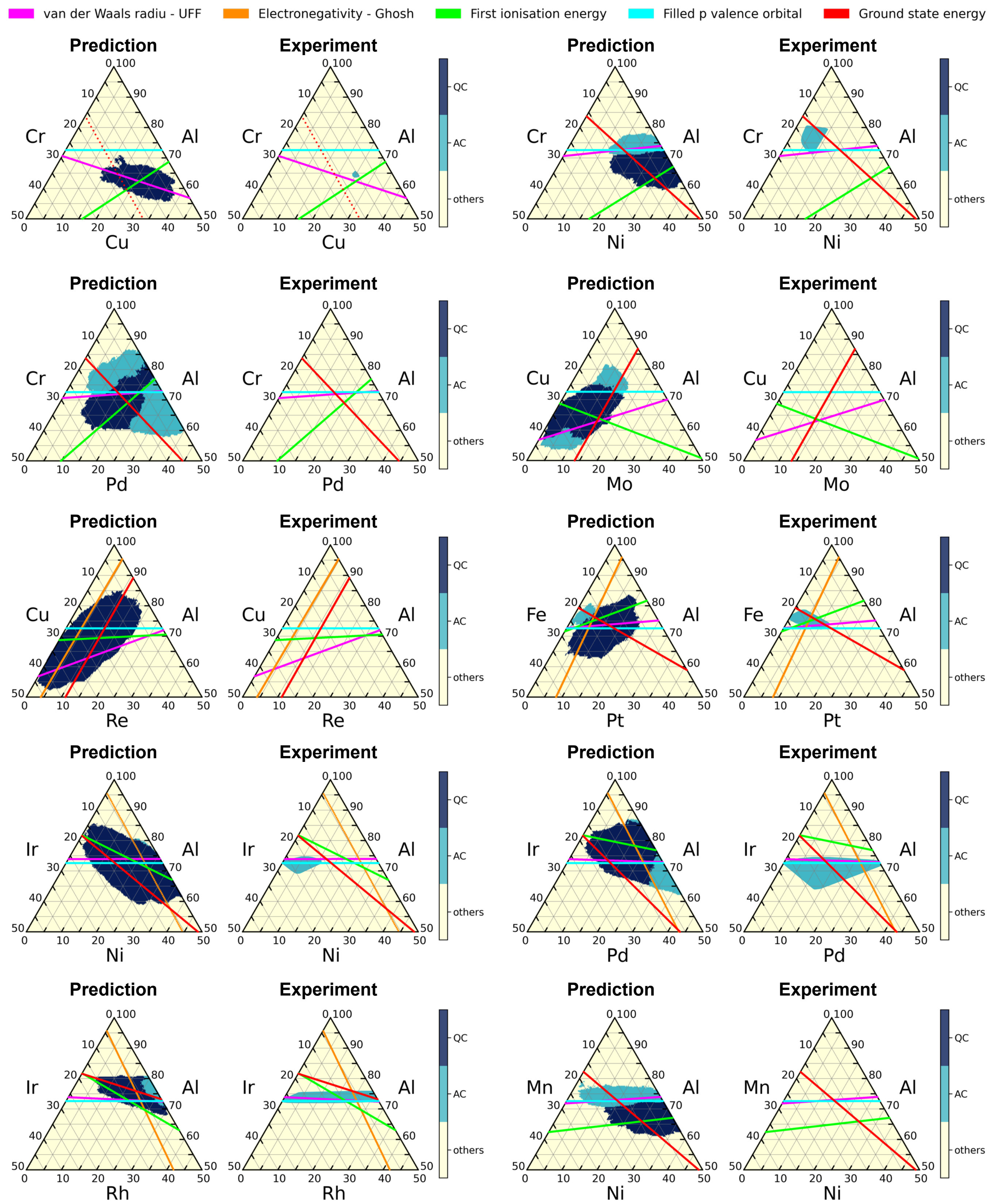
van der Waals radiu - UFF
Electronegativity - Ghosh
First ionisation energy
Filled p valence orbital
Ground state energy
Prediction
Experiment
Cr
Al
Cu
QC
AC
others
Prediction
Experiment
Cr
Al
Ni
QC
AC
others
Prediction
Experiment
Cr
Al
Pd
QC
AC
others
Prediction
Experiment
Cu
Al
Mo
QC
AC
others
Prediction
Experiment
Cu
Al
Re
QC
AC
others
Prediction
Experiment
Fe
Al
Pt
QC
AC
others
Prediction
Experiment
Ir
Al
Ni
QC
AC
others
Prediction
Experiment
Ir
Al
Pd
QC
AC
others
Prediction
Experiment
Ir
Al
Rh
QC
AC
others
Prediction
Experiment
Mn
Al
Ni
QC
AC
others

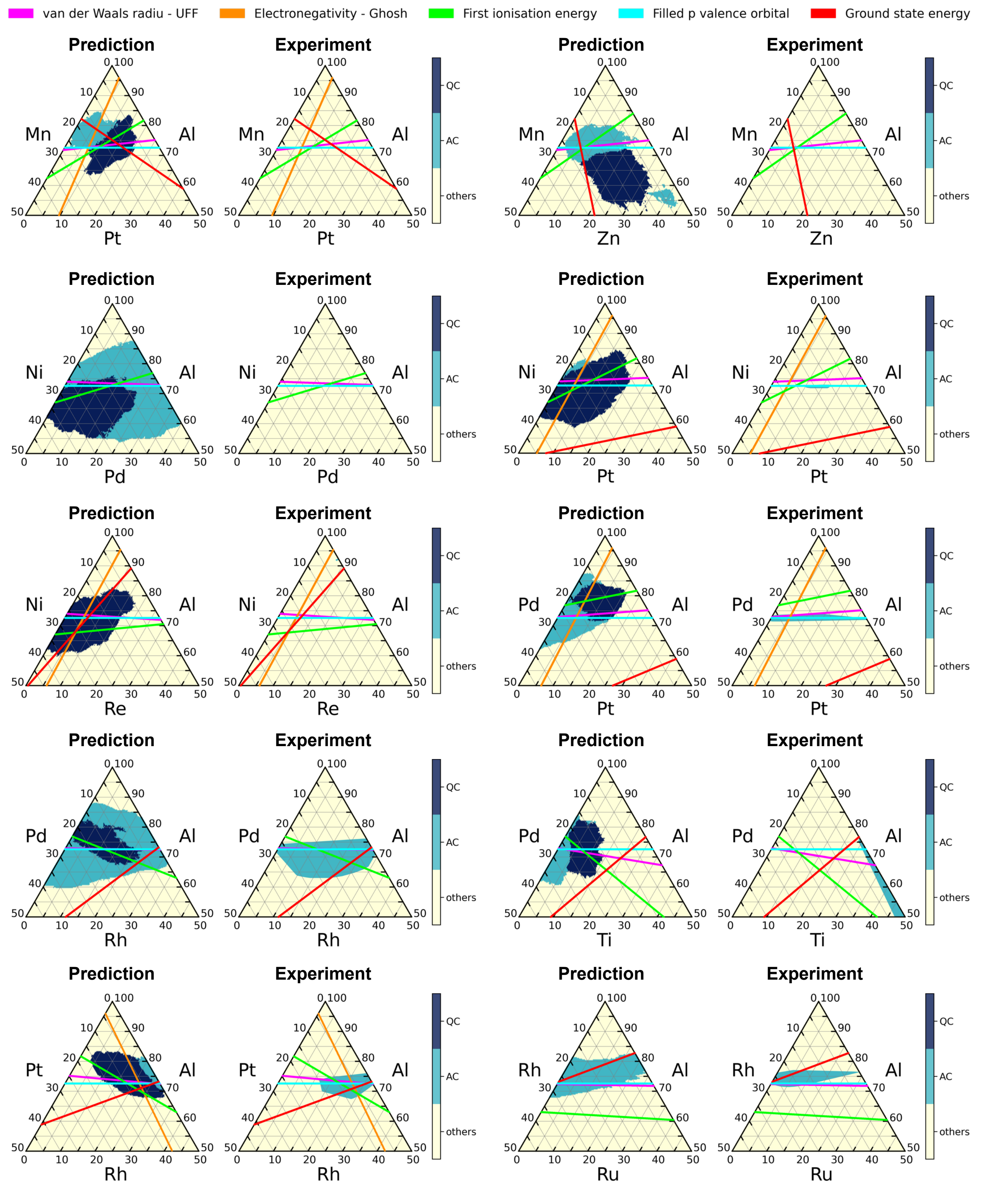

van der Waals radiu - UFF
Electronegativity - Ghosh
First ionisation energy
Filled p valence orbital
Ground state energy
Prediction
Experiment
Mn
Al
Pt
Zn
QC
AC
others
Ni
Pd
Re
Rh
Ti
Ru

References

[1] S. Walter, S. Deloudi, *Crystallography of Quasicrystals*, volume 126 of *Springer Series in Materials Science*, Springer Berlin Heidelberg, Berlin, Heidelberg, **2009**.

[2] Xenonpy platform, https://github.com/yoshida-lab/XenonPy, Accessed: 2021-4-30.

[3] J. C. Slater, *J. Chem. Phys.* **1964**, *41*, 10 3199.

[4] mendeleev – a python resource for properties of chemical elements, ions and isotopes, ver. 0.3.6, https://github.com/lmmentel/mendeleev, Accessed: 2021-4-30.

[5] M. Rahm, R. Hoffmann, N. W. Ashcroft, *Chem. Eur. J.* **2016**, *22*, 41 14625.

[6] M. Rahm, R. Hoffmann, N. W. Ashcroft, *Chem. Eur. J.* **2017**, *23*, 16 4017.

[7] J. Vogt, S. Alvarez, *Inorg. Chem.* **2014**, *53*, 17 9260.

[8] J. Meija, T. B. Coplen, M. Berglund, W. A. Brand, P. De Bièvre, M. Gröning, N. E. Holden, J. Irrgeher, R. D. Loss, T. Walczyk, T. Prohaska, *Pure Appl. Chem.* **2016**, *88*, 3 265.

[9] S. P. Ong, W. D. Richards, A. Jain, G. Hautier, M. Kocher, S. Cholia, D. Gunter, V. L. Chevrier, K. A. Persson, G. Ceder, *Comput. Mater. Sci.* **2013**, *68* 314.

[10] K. T. Tang, J. M. Norbeck, P. R. Certain, *J. Chem. Phys.* **1976**, *64*, 7 3063.

[11] X. Chu, A. Dalgarno, *J. Chem. Phys.* **2004**, *121*, 9 4083.

[12] B. Cordero, V. Gómez, A. E. Platero-Prats, M. Revés, J. Echeverría, E. Cremades, F. Barragán, S. Alvarez, *Dalt. Trans.* **2008**, 2832.

[13] P. Pyykkö, M. Atsumi, *Chem. Eur. J.* **2009**, *15*, 46 12770.

[14] P. Pyykkö, M. Atsumi, *Chem. Eur. J.* **2009**, *15*, 1 186.

[15] P. Pyykkö, S. Riedel, M. Patzschke, *Chem. Eur. J.* **2005**, *11*, 12 3511.

[16] P. Schwerdtfeger, J. K. Nagle, *Mol. Phys.* **2019**, *117*, 9-12 1200.

[17] L. Ward, A. Agrawal, A. Choudhary, C. Wolverton, *npj Comput. Mater.* **2016**, *2*, July 1.

[18] W. M. Haynes, *CRC Handbook of Chemistry and Physics, 95th Edition*, Oakville : CRC Press, 95th ed edition, **2014**.

[19] T. Andersen, *Phys. Rep.* **2004**, *394*, 4-5 157.

[20] J. B. Mann, T. L. Meek, L. C. Allen, *J. Am. Chem. Soc.* **2000**, *122*, 12 2780.

[21] J. B. Mann, T. L. Meek, E. T. Knight, J. F. Capitani, L. C. Allen, *J. Am. Chem. Soc.* **2000**, *122*, 21 5132.

[22] D. C. Ghosh, *J. Theor. Comput. Chem.* **2005**, *04*, 01 21.

[23] J. E. Saal, S. Kirklin, M. Aykol, B. Meredig, C. Wolverton, *JOM* **2013**, *65*, 11 1501.

[24] S. Kirklin, J. E. Saal, B. Meredig, A. Thompson, J. W. Doak, M. Aykol, S. Rühl, C. Wolverton, *npj Comput. Mater.* **2015**, *1*, November.

[25] M. W. Gaultois, T. D. Sparks, C. K. H. Borg, R. Seshadri, W. D. Bonificio, D. R. Clarke, *Chem. Mater.* **2013**, *25*, 15 2911.

[26] A. Belsky, M. Hellenbrandt, V. L. Karen, P. Luksch, *Acta Crystallogr. Sect. B Struct. Sci.* **2002**, *58*, 3 364.

[27] R. Allmann, R. Hinek, *Acta Crystallogr. Sect. A Found. Crystallogr.* **2007**, *63*, 5 412.

[28] D. Zagorac, H. Muller, S. Ruehl, J. Zagorac, S. Rehme, *J. Appl. Crystallogr.* **2019**, *52* 918.

[29] D. G. Pettifor, *Solid State Commun.* **1984**, *51*, 1 31.

[30] P. Villars, K. Cenzual, J. Daams, Y. Chen, S. Iwata, *J. Alloys Compd.* **2004**, *367*, 1-2 167.

[31] Material-agnostic platform for informatics and exploration, https://wolverton.bitbucket.io, Accessed: 2021-4-30.

[32] Electron configuration of the elements, https://periodictable.com/Properties/A/ElectronConfigurationString.v.html, Accessed: 2021-4-30.

[33] S. Alvarez, *Dalt. Trans.* **2013**, *42*, 24 8617.

[34] N. L. Allinger, X. Zhou, J. Bergsma, *Theochem J. Mol. Struct.* **1994**, *312*, 1 69.

[35] A. K. Rappe, C. J. Casewit, K. S. Colwell, W. A. Goddard, W. M. Skiff, *J. Am. Chem. Soc.* **1992**, *114*, 25 10024.
